# *Drosophila* SPARC collagen IV chaperone-like activity essential for development is unique to the fat body

**DOI:** 10.1016/j.isci.2025.112111

**Published:** 2025-02-27

**Authors:** Samuel Delage, Arya Zadhoosh, William You, Theodore Joseph Brown, Maurice Joseph Ringuette

**Affiliations:** 1 Department of Cell and Systems Biology, University of Toronto, Toronto, ON M5S 3G5, Canada; 2 Department of Obstetrics and Gynaecology, University of Toronto, Toronto, ON M5G 1E2, Canada; 3 Lunenfeld-Tanenbaum, Research Institute at Sinai Health Systems, Toronto, ON M5G 1X5, Canada

**Keywords:** Cell biology, Developmental biology

## Abstract

*Drosophila* fat body-derived SPARC acts as a chaperone for collagen IV (Col(IV)), enabling their diffusion and incorporation into distal tissue basement membranes (BMs). Disruption of SPARC or Col(IV) production by the fat body is lethal, despite expression by other tissues such as imaginal discs. Wing disc-derived SPARC does not associate with Col(IV) in BMs and is not essential for survival. We show that differential association of fat body- and wing disc-derived SPARC with Col(IV) is not due to differences in SPARC glycosylation nor to the absence of SPARC and Col(IV) co-expression. Further, we demonstrate that SPARC domain II/III produced by the fat body is sufficient for Col(IV) diffusion to both proximal and distal BMs, and rescues lethality associated with loss of SPARC. However, SPARC domain II/III does not diffuse beyond the hemolymph. Thus, the essential Col(IV) chaperone-like activity specific to fat body-derived SPARC is not required beyond the hemolymph.

## Highlights

• Differences in SPARC glycosylation do not impact its association with collagen IV
• SPARC from wing, eye, or leg discs does not associate with collagen IV at BMs
• Fat body SPARC domain II/III rescues lethality associated with the loss of SPARC
• Fat body SPARC domain II/III enables the diffusion of collagen IV to distal BMs

## Graphical abstract

**Figure:**
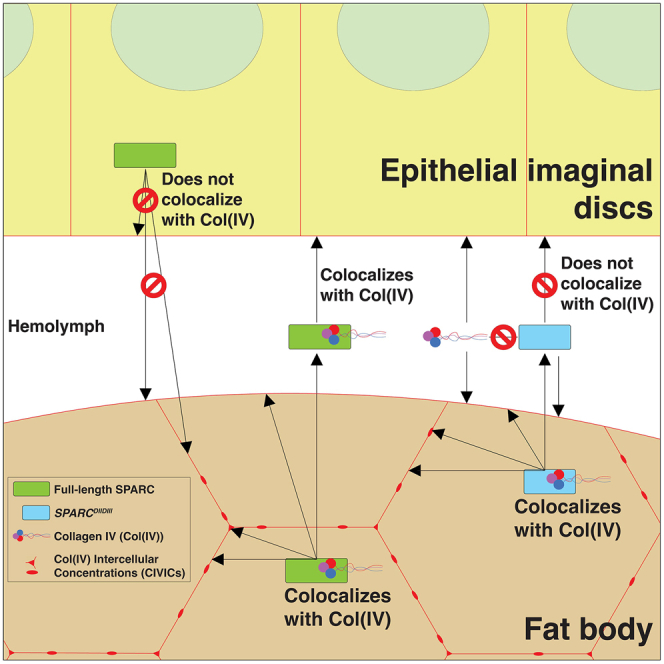

## Introduction

Basement membranes (BMs) are thin, sheet-like extracellular matrices (ECMs) that form at the basal surface of epithelial, endothelial, and lymphatic cells, and surround specialized cells such as muscle, Schwann, and adipose cells. As such, BMs play indispensable tissue-specific roles in the development, physiology, and regenerative capacity of all multicellular organisms. Tissue- or context-dependent functions of BMs include establishing tissue epithelialization and compartmentalization, providing attachment sites for cells, and imparting mechanical strength and shape to tissues. BMs also regulate cell signaling by binding to a variety of transmembrane receptors and growth factors that transduce intracellular signals.^1^^,^^2^^,^^3^^,^^4^ Consequently, mutations or dysregulation in the expression, assembly, and remodeling of BM constituents can lead to significant disruptions in development and tissue homeostasis that underlie multiple pathologies.^4^

BMs are composed of four universal core components: laminins, collagen IV (Col(IV)), perlecan, and nidogens, which are produced by various cell types in a tissue-specific manner. The initial step in the assembly of most BMs involves laminin anchoring and polymerization at cell surface receptors, most notably dystroglycans and integrins.^5^^,^^6^ Col(IV), which constitutes up to 50% of the BM mass and imparts tensile strength, then integrates as a second network, anchoring onto the laminin scaffold and integrins. Perlecan, a large BM heparan sulfate proteoglycan, is then recruited and integrated,^7^^,^^8^ where it functions to modulate the tensile strength of the BM.^8^ Nidogen, also referred to as entactin, is a glycoprotein thought to act as a linker/adapter protein in BMs by promoting the stability of ternary complexes consisting of laminin, Col(IV), and perlecan.^9^

Col(IV) protomers have an innate ability to self-polymerize; thus, the precocious assembly of Col(IV) networks must be prevented to allow proper Col(IV) network integration onto the integrin-tethered laminin network. This is of particular importance for invertebrates whereby Col(IV) and other core components are synthesized by specific tissues and must diffuse to and assemble into BMs at distal sites. Evidence from our laboratory and others indicate non-core components contribute to BM assembly, remodeling, and function, often in a tissue-specific manner.^4^^,^^6^ We have previously shown the non-core BM component SPARC (Secreted Protein, Acidic, Rich in Cysteine) is required for the proper assembly of Col(IV) into BMs in *Drosophila*.^10^^,^^11^ Loss of fat body-derived SPARC leads to intracellular retention of Col(IV) and a cell-autonomous aberrant, fibrotic-like accumulation of Col(IV) in BMs surrounding fat body adipocytes, resulting in lethality at the second instar larval stage. Moreover, the diffusion of Col(IV) from the fat body to distal sites is prevented in the absence of SPARC.^8^^,^^10^^,^^12^^,^^13^ This has provided evidence to support the paradigm-shifting concept that SPARC functions as an essential intracellular and extracellular chaperone-like glycoprotein for Col(IV) first proposed by Martinek et al.^14^ In further support of this chaperone-like activity of SPARC, we reported that SPARC and Col(IV) first colocalize in the trans-Golgi network just prior to their secretion and that mutating the collagen-binding epitopes of SPARC prevents its colocalization with Col(IV) in BMs.^11^

Mammals express two glycoforms of SPARC, bone-derived SPARC and platelet-derived SPARC, which differ in both their N-glycosylation sugar groups and their ability to bind collagens I, III, IV, and V.^15^ Collagen binding simulation studies suggest the N-glycosylation sugar groups of platelet-derived SPARC hinder collagen binding whereas those of bone-derived SPARC enable collagen binding.^16^

Similar to mammalian SPARC, our laboratory has shown the ability of *Drosophila* SPARC to colocalize with Col(IV) in BMs is also dependent on its tissue of origin. Fat body-derived SPARC concentrates within BMs at both proximal and distal sites, whereas SPARC produced by the wing disc epithelium fails to colocalize with Col(IV) at either the wing discs or fat body BM, despite the fact that it is able to diffuse to the latter.^11^ In the present study, we sought to determine the underlying cause of tissue-specific differences in the ability for SPARC to colocalize with Col(IV). Our results indicate the functional differences between fat body-derived SPARC and imaginal disc-derived SPARC are due to tissue-specific properties rather than glycosylation or co-expression with Col(IV). Our studies further indicate that the chaperone-like activity of SPARC, which enables Col(IV) to diffuse to and integrate into BMs at distal sites is not required beyond the delivery of Col(IV) to the hemolymph.

## Results

### Fat body-derived SPARC is not the underlying cause preventing wing disc-derived SPARC from colocalizing with Col(IV) in the fat body

A possible explanation for the lack of colocalization between wing disc-derived SPARC and Col(IV) in the fat body BM may be due to competition by the high levels of SPARC produced by the fat body. Due to their being polyploid, fat body adipocytes produce and secrete greater amounts of ECM components compared to diploid imaginal disc epithelial cells.^8^^,^^17^ To test for the possibility that wing disc-derived SPARC is unable to colocalize with Col(IV) in the fat body BM due to Col(IV) being saturated by fat body-derived SPARC, it was necessary to simultaneously knock down endogenous SPARC production in the fat body while expressing SPARC::HA in the wing discs. While the most parsimonious approach would be to express wing disc-derived SPARC::HA in a *SPARC*-null background, this is not feasible given the lethality associated with the loss of SPARC.^11^ We thus devised a strategy whereby we expressed SPARC::HA in the wing discs and *SPARC*^*RNAi*^ to knock down SPARC in the fat body.

It was necessary to first establish the efficacy and time course for SPARC knockdown. We used a *Drosophila* fly line where Gal4 was expressed under control of the endogenous SPARC promoter. These flies were crossed to *SPARC*^*RNAi*^ flies. We then crossed the progeny to a fly line where a temperature-sensitive form of Gal80 (Gal80^TS^), a Gal4 suppressor, was expressed under the ubiquitous tubulin promoter. Gal80^TS^ is active at 18°C but conformationally inactive at 29°C. Activation of *SPARC*^*RNAi*^ by transferring larvae to 29°C resulted in a gradual reduction in SPARC levels to non-detectable by 18 h (Figure S1). This delayed knockdown enables residual SPARC mRNA to be transcribed and protein to be secreted following activation of *SPARC*^*RNAi*^.

**Figure 1:**
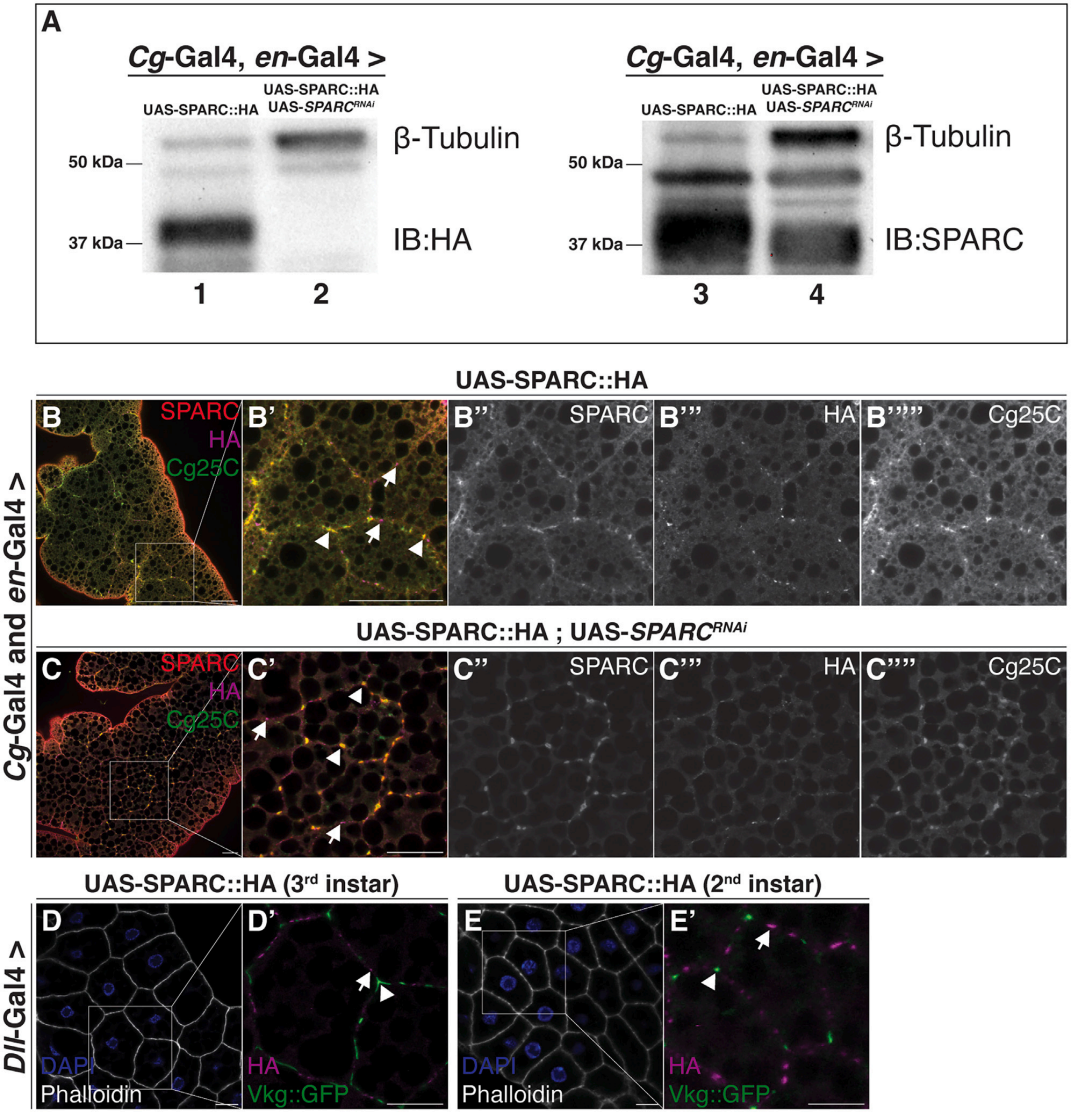
Saturation of Col(IV) in the larval fat body is not the underlying cause for the lack of colocalization between wing disc-derived SPARC and Col(IV) (A) Western blot showing levels of SPARC::HA (left) (∼40 kDa) and endogenous SPARC (right) (∼37 kDa) in third-instar larvae driving expression of SPARC::HA only (Lanes 1 and 3) and SPARC::HA, *SPARC*^*RNAi*^ (Lanes 2 and 4). β-tubulin (∼55 kDa) was used as a loading control. (B and C) Endogenous SPARC (red), HA (magenta), and Col(IV) (green) immunostaining of third instar larval fat bodies from larvae expressing fat body- and wing disc-derived SPARC::HA (B–B”’’) and SPARC::HA, *SPARC*^*RNAi*^ (C–C”’’). White box indicates the zoomed in region. Arrowheads indicate endogenous SPARC and Col(IV) colocalization. Arrows indicate SPARC::HA that does not associate with Col(IV). (D–E) Third instar (D) and second instar (E) larval fat bodies from larvae expressing leg disc-derived SPARC::HA stained for actin (phalloidin—white) and chromatin (DAPI—blue) and immunostained with HA (magenta). Vkg::GFP (green) was used to visualize Vkg, the α2 chain of *Drosophila* Col(IV). Arrowheads indicate CIVICs. Arrows indicate SPARC::HA that does not associate with Col(IV). Scale bars represent 25 μm. Data shown are representative of a minimum of three biological replicates. See also Figure S1 and S2.

*Cg-Gal4*, a fat body- and hemocyte-specific driver line, was used to drive expression of *SPARC*^*RNAi*^ starting at early embryogenesis, whereas *en*-Gal4 was used to drive SPARC::HA expression in the posterior compartment of the wing discs at the third instar.^18^ Consistent with our *SPARC*^*RNAi*^ time course experiment, the majority of endogenous SPARC derived from the fat body is knocked down by the early third instar larval stage and a proportion of SPARC::HA derived from the wing discs was present. Western blot analyses showed a decrease in the band intensity for endogenous SPARC and SPARC::HA, albeit present (Figure 1A). Despite the knockdown of fat body-derived SPARC, SPARC::HA did not colocalize with Col(IV) in the larval fat body (Figures 1B–1C’’’’). In the absence of *SPARC*^*RNAi*^, immunostaining of fat bodies in which the larvae expressed SPARC::HA in both the fat body and wing discs, showed both endogenous SPARC and HA-tagged SPARC colocalizing with Col(IV). Consistent with previous studies,^11^ a subset of SPARC::HA localized pericellularly within the fat body, distinct from the localization of Col(IV) (Figures 1B–1B’’’’). Immunostaining of fat bodies in which endogenous SPARC was simultaneously knocked down in the fat body and SPARC::HA was expressed in the wing discs showed endogenous SPARC staining colocalized with Col(IV). SPARC::HA staining decreased in intensity but remained localized pericellularly within the fat body, distinct from the localization of Col(IV) (Figures 1C–1C’’’’).

We previously reported the synthesis of SPARC and Col(IV) may be greater at the second instar compared to the third instar,^11^ raising the possibility that by the third instar larval stage, Col(IV) in the fat body BM may already be saturated by fat body derived SPARC, thereby preventing wing disc-derived SPARC from colocalizing with Col(IV). Thus, to further test whether wing disc-derived SPARC is unable to colocalize with Col(IV) as a result of Col(IV) being saturated by fat body-derived SPARC, we stained the fat bodies of second instar larvae. In the context of the wing discs, the expression of *en*-Gal4 is restricted to its posterior compartment and the third instar developmental stage, thereby preventing us from using *en*-Gal4 to drive expression of wing disc-derived SPARC at the second instar. Therefore, the leg disc driver line *Dll*-Gal4 was used to drive expression of SPARC::HA in the leg discs, which phenocopies the pericellular localization of wing disc-derived SPARC::HA in the fat body of third instar larvae (data presented in Figure 3). Immunostaining of leg disc-derived SPARC::HA at the second instar shows a similar phenotype to leg disc-derived SPARC::HA at the third instar, whereby SPARC::HA staining does not colocalize with Col(IV), visualized by a functional GFP-trap fusion of the α2 chain *Vkg* (Vkg::GFP) (Figures 1D–1E′). Although immunostaining of wild-type larval fat bodies not expressing SPARC::HA using a combination of both the mouse anti-HA primary and anti-mouse secondary antibodies showed some pericellular HA staining, the punctae were fewer and fainter (Figure S2). Collectively, these data indicate the inability for epithelial wing disc-derived SPARC to colocalize with Col(IV) in the fat body is not likely due to Col(IV) being saturated by polyploid fat body adipocyte-derived SPARC.

### N-glycosylation is not required for fat body-derived SPARC to colocalize with Col(IV) in BMs

**Figure 2:**
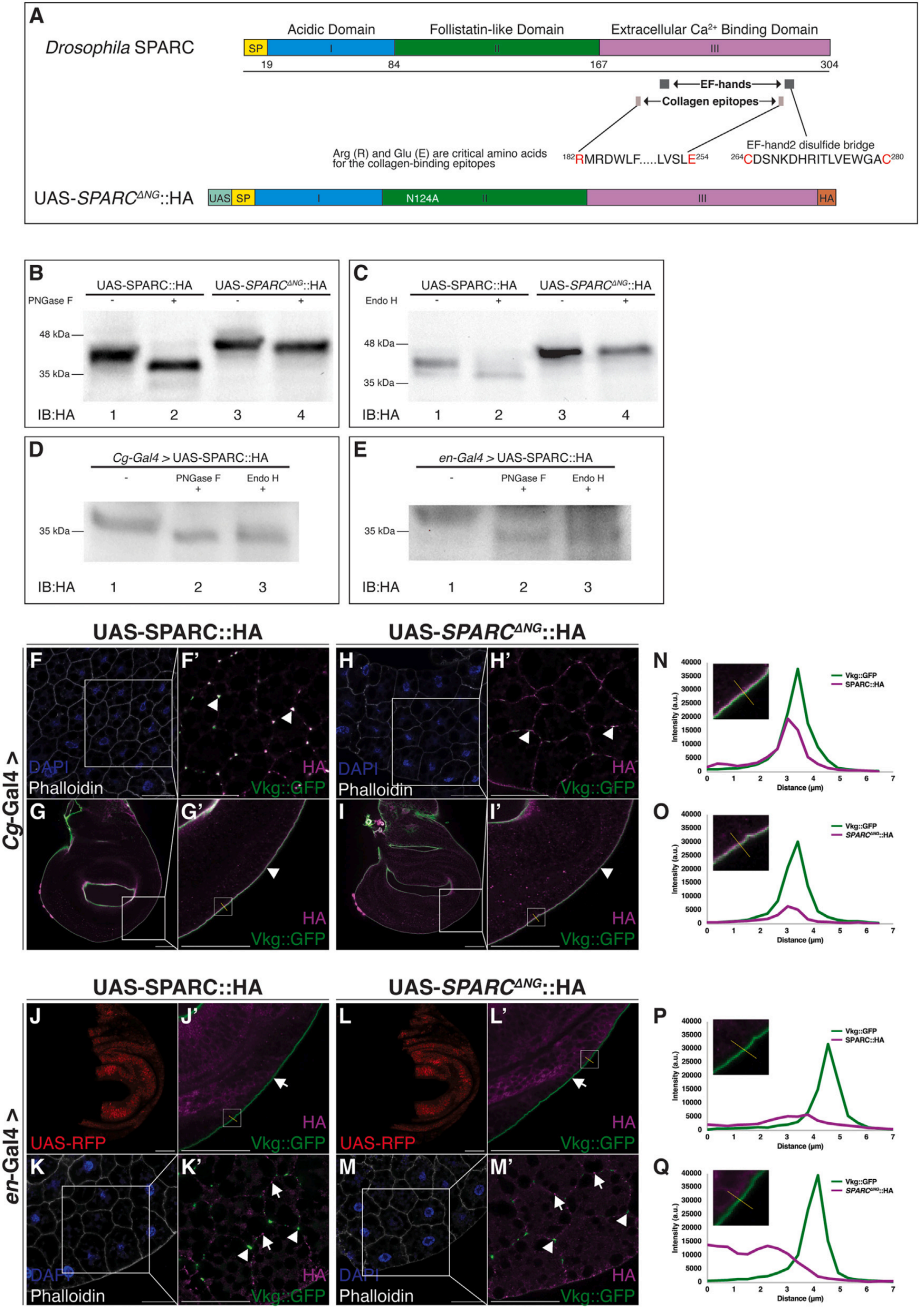
Removal of N-glycosylation does not enable wing disc-derived SPARC to colocalize with Col(IV) (A) Schematic representation of transgenic SPARC constructs generated using gateway cloning technology. The schematic indicates the UAS promoter (UAS; teal), a signal peptide (SP; yellow), SPARC domain I (blue), SPARC domain II (green), SPARC domain III (purple), and C-terminal HA tag (orange). Amino acids predicted to be critical for collagen binding and cysteines involved in the formation of the disulfide bridge in EF-hand2 are highlighted in red. The amino acid substitutions for the N-glycosylation acceptor site of SPARC are indicated in white within domain II. (B and C) Western blot analysis showing the shift in molecular weight of untreated (−) and peptide-N-glycosidase F (PNGase F)-treated (B) or endoglycosidase H (Endo H)-treated (C) (+) fly lysates extracted from flies expressing SPARC::HA (Lanes 1 and 2) and *SPARC*^*ΔNG*^::HA (Lanes 3 and 4). (D and E) Western blot analysis showing the shift in molecular weight of untreated (−), PNGase F-treated (+) or Endo H treated (+) fat body- (D) or wing disc- (E) derived SPARC::HA. (F and G) Immunostaining of third instar larval fat bodies (F–F’ and H–H’) and wing discs (G–G’ and I–I’) from larvae expressing fat body-derived SPARC::HA (F–G) and *SPARC*^*ΔNG*^::HA (H and I). Fat bodies were stained for actin (phalloidin—white) and chromatin (DAPI—blue) and immunostained with HA (magenta). Wing discs were immunostained with HA (magenta). Vkg:GFP (green) was used to visualize Vkg. White box indicates the zoomed in region. Arrowheads indicate HA and Vkg::GFP colocalization. (J–M) Immunostaining of third instar larval wing discs (J–J’ and L–L’) and fat bodies (K–K’ and M–M’) from larvae expressing wing disc-derived SPARC::HA (J and K) and *SPARC*^*ΔNG*^::HA (L and M). Wing discs were immunostained with HA (magenta). Fat bodies were stained for actin (phalloidin—white) and chromatin (DAPI—blue) and immunostained with HA (magenta). Vkg::GFP (green) was used to visualize Vkg. White box indicates the zoomed in region. Arrowheads indicate Vkg::GFP signal in CIVICs. Arrows indicate SPARC::HA and *SPARC*^*ΔNG*^::HA that does not associate with Vkg::GFP. Scale bars represent 25 μm. Data shown are representative of a minimum of three biological replicates. (N–Q) Intensity plot profiles for Vkg::GFP (green) and fat body or wing disc-derived SPARC::HA (N and P, respectively) and *SPARC*^*ΔNG*^::HA (O and Q, respectively) (magenta) along the yellow solid line. Cutout in each plot shows the magnified image and region of the discs from which the intensity plots were generated.

Mammalian studies indicate that removal of N-glycosylation increases the binding affinity of SPARC for fibril-forming collagens type I, III, and V, and importantly, Col(IV).^19^^,^^20^^,^^21^ We thus generated a construct in which the single predicted N-glycosylation acceptor site of SPARC was mutated by substituting the asparagine residue at position 124 to alanine (Asn124Ala) (*SPARC*^*ΔNG*^::HA) (Figure 2A) to determine if removing the capacity for N-glycosylation enables wing disc-derived SPARC to colocalize with Col(IV) in BMs.

To confirm that this sequence-verified mutation resulted in the loss of N-glycosylation, fly lysates were treated with peptide-N-glycosidase F (PNGase F), which cleaves the innermost N-glycosidic bond, thus removing N-linked oligosaccharides from glycoproteins. Western blot analyses using HA antibody show SPARC::HA lysate digested with PNGase F resulted in an expected decreased molecular weight of approximately 2–4 kDa (Figure 2B, lane 2) compared to undigested lysate (Figure 2B, lane 1). In contrast, digestion of *SPARC*^*ΔNG*^::HA lysate with PNGase F showed little or no shift in molecular weight compared to the undigested lysate (Figure 2B, lane 3 and lane 4). These findings suggest that removing the N-glycosylation acceptor site at position 124 of SPARC successfully prevents N-glycosylation of *SPARC*^*ΔNG*^::HA.

To test for the possibility of different SPARC glycoforms being expressed in *Drosophila*, fly lysates were treated with endoglycosidase H (Endo H), a recombinant glycosidase, which cleaves within the chitobiose core of high mannose and some hybrid oligosaccharides from N-linked glycoproteins, removing only a subset of N-linked oligosaccharides from glycoproteins. Similar to PNGase F digestion, western blot analyses using an HA antibody showed SPARC::HA lysate digested with Endo H resulted in a comparative decrease in molecular weight of approximately 2–4 kDa (Figure 2C, lane 2) compared to undigested lysate (Figure 2C, lane 1). In contrast, digestion of *SPARC*^*ΔNG*^::HA lysate with Endo H showed little or no change in molecular weight compared to the undigested lysate (Figure 2C, lane 3 and lane 4). To further test whether fat body- and wing disc-derived SPARC have different N-glycosylation patterns, fat body- and wing disc-derived SPARC::HA were treated with PNGase F or Endo H. Immunoblotting against ΗΑ showed the SPARC::HA band from either the fat body or wing discs had similar molecular weights (Figures 2D and 2E). Collectively, these data suggest mutating the asparagine residue at position 124 to an alanine successfully removed the single N-glycosylation acceptor site of SPARC and that there are no overt differences in the N-glycosylation patterns of fat body- and wing disc-derived SPARC.

To examine the impact of removing the N-glycosylation acceptor site of SPARC on its ability to colocalize with Col(IV), SPARC::HA and *SPARC*^*ΔNG*^::HA were expressed in the fat body in a wild-type background using *Cg*-Gal4. As expected, fat body-derived SPARC::HA and *SPARC*^*ΔNG*^::HA localized to fat body adipocyte cell borders and within Col(IV) Intercellular Concentrations (CIVICs) (Figures 2F–2H’).^22^ The merged images between Vkg::GFP and anti-HA staining showed overlap (Figure 2H’), indicating that as in vertebrates,^20^^,^^21^ removal of N-glycosylation does not prevent the association between SPARC and Col(IV) in *Drosophila*. As the fat body is the main source of BM components during larval development, we determined if removing the N-glycosylation acceptor site of SPARC affects its ability to colocalize with Col(IV) at distal sites, such as the wing discs. Fat body-derived SPARC::HA and *SPARC*^*ΔNG*^::HA both localized to the wing disc BM (Figures 2G–2G′ and 2I–2I′). The merged images between Vkg::GFP and anti-HA showed a complete overlap at the wing disc BM (Figures 2G’, 2I’, 2N, and 2O). Collectively, these data indicate that removing the N-glycosylation acceptor site of SPARC does not impair the ability of fat body-derived SPARC to colocalize with Col(IV) at proximal or distal sites.

### Removal of N-glycosylation does not enable wing disc-derived SPARC to colocalize with Col(IV) in BMs

We determined if removing the N-glycosylation acceptor of wing disc-derived SPARC impacts its ability to colocalize with Col(IV) by expressing SPARC::HA and *SPARC*^*ΔNG*^::HA in the wing discs in a wild-type background using *en*-Gal4. Confirming our previous report,^11^ wing disc-derived SPARC::HA did not localize to the wing disc BM (Figures 2J–2J’, 2P). Surprisingly, removal of the N-glycosylation acceptor site of SPARC did not impart an ability for wing disc-derived SPARC to colocalize with Col(IV), in contrast to what has been reported for mammalian SPARC. The merged images between Vkg::GFP and anti-HA showed no overlap (Figures 2J’, 2L’, 2P, and 2Q). Rather, SPARC::HA and *SPARC*^*ΔNG*^::HA localized within the posterior compartment of wing discs, similar to the distribution of UAS-RFP (Figures 2J–2J’ and 2L–2L’). We next determined if removing the N-glycosylation acceptor of wing disc-derived SPARC affects its ability to colocalize with Col(IV) in the fat body BM. Neither wing disc-derived SPARC::HA nor *SPARC*^*ΔNG*^::HA localized to the fat body BM (Figures 2K’ and 2M’). Rather, SPARC::HA and *SPARC*^*ΔNG*^::HA localized to adipocyte borders within the fat body, distinct from Col(IV) (Figures 2K’ and 2M’). Collectively, these data show that tissue-specific differences in N-glycosylation of SPARC do not determine the ability of SPARC to associate with Col(IV) in the larval fat body.

### Eye and leg disc-derived SPARC do not colocalize with Col(IV) in BMs

We previously demonstrated the Col(IV) chaperone-like activity of SPARC first occurs in the secretory pathway, as evidenced by the colocalization of SPARC and Col(IV) in the trans-Golgi network of hemocyte-like S2 cells.^11^ Furthermore, Col(IV) is retained intracellularly and fails to diffuse to distal tissues in the absence of SPARC or when the collagen-binding domains of SPARC have been mutated.^11^ These findings demonstrate the Col(IV) chaperone-like activity of SPARC is required for proper Col(IV) assembly in BMs, and raise the possibility SPARC and Col(IV) need to be co-secreted to colocalize in BMs. Unlike the wing discs, which express SPARC but not Col(IV),^23^^,^^24^^,^^25^ the eye and leg discs produce both SPARC and Col(IV), similar to the fat body.^26^ Therefore, we used these tissues to determine if SPARC and Col(IV) need to be co-expressed to colocalize in BMs.

**Figure 3:**
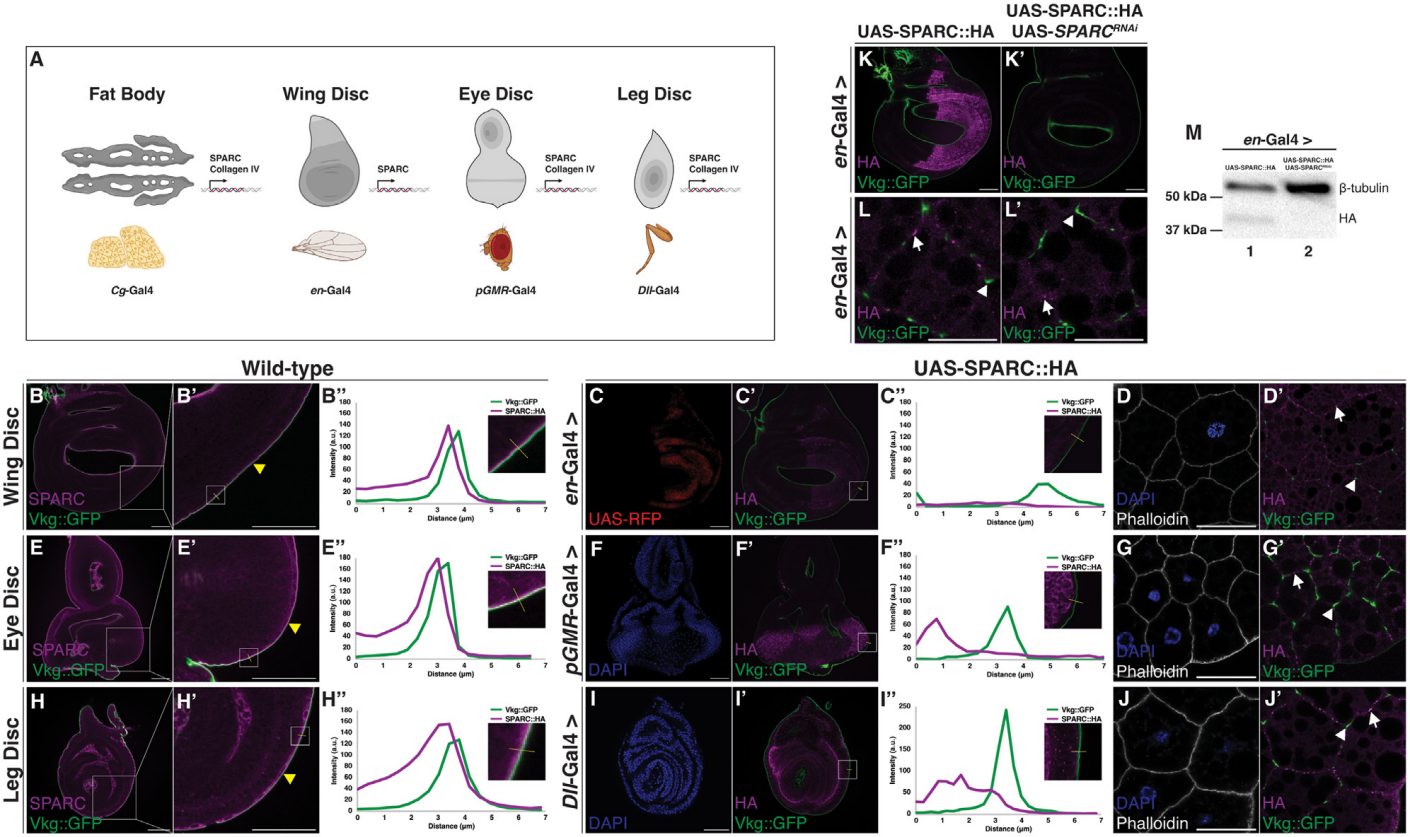
Epithelial imaginal disc tissues express a SPARC protein product that does not colocalize with Col(IV) in their respective BMs nor in the fat body BM (A) Schematic highlighting tissue-specific differences in the production of SPARC and Col(IV) in the fat body, wing, eye, and leg discs. (B–D) Images of wild type wing discs immunostained for endogenous SPARC (magenta) (B–B’) and wing discs (C–C’) or fat bodies (D–D’) dissected from larvae expressing wing disc-derived SPARC::HA immunostained for HA (magenta). Fat bodies were stained for actin (phalloidin—white) and chromatin (DAPI—blue). Vkg::GFP (green) was used to visualize Vkg. White box indicates the zoomed in region. Yellow arrowheads indicate SPARC and Vkg::GFP colocalization. White arrowheads indicate Vkg::GFP signal. Arrows indicate SPARC::HA that does not associate with Vkg::GFP. (E–G) Images of wild type eye discs immunostained for endogenous SPARC (magenta) (E–E’) and eye discs (F–F’) or fat bodies (G–G’) dissected from larvae expressing eye disc-derived SPARC::HA immunostained for HA (magenta). Fat bodies were stained for actin (phalloidin—white) and chromatin (DAPI—blue). Vkg::GFP (green) was used to visualize Vkg. White box indicates the zoomed in region. White arrowheads indicate Vkg::GFP signal. Arrows indicate SPARC::HA that does not associate with Vkg::GFP. (H–J) Images of wild-type leg discs immunostained for endogenous SPARC (magenta) (H–H’) and leg discs (I–I’) or fat bodies (J–J’) dissected from larvae expressing leg disc-derived SPARC::HA immunostained for HA (magenta). Fat bodies were stained for actin (phalloidin—white) and chromatin (DAPI—blue). Vkg::GFP (green) was used to visualize Vkg. White box indicates the zoomed in region. White arrowheads indicate Vkg::GFP signal. Arrows indicate SPARC::HA that does not associate with Vkg::GFP. Intensity plot profiles for Vkg::GFP (green) and SPARC or SPARC::HA (magenta) along the yellow solid line (B”, C”, E”, F”, H”, and I”). Cutout in each plot shows the magnified image and region of the discs from which the intensity plots were generated. (K) Immunostaining of HA (magenta) in wing discs expressing SPARC::HA (K) or SPARC::HA, *SPARC*^*RNAi*^ (K’). Vkg::GFP (green) was used to visualize Vkg. (L) Immunostaining of HA (magenta) in third instar larval fat bodies dissected from larvae expressing wing disc-derived SPARC::HA (L) or SPARC::HA, *SPARC*^*RNAi*^ (L’). Vkg::GFP (green) was used to visualize Vkg. White arrowheads indicate Vkg::GFP signal. Arrows indicate SPARC::HA that does not associate with Vkg::GFP. Scale bars represent 25 μm. (M) Western blot showing levels of SPARC::HA (∼37 kDa) generated in third-instar larvae driving expression of SPARC::HA only (Lane 1) or SPARC::HA, *SPARC*^*RNAi*^ (Lanes 2). β-tubulin (∼55 kDa) was used as a loading control. Data shown are representative of a minimum of three biological replicates. See also Figures S3 and S4.

We first determined if eye disc-derived SPARC localized to the eye disc BM. SPARC::HA was expressed in the eye discs using *pGMR*-Gal4, which drives expression in the eye disc posterior to the morphogenetic furrow. To compare the localization of eye disc-derived SPARC::HA to wild-type SPARC, anti-SPARC immunostaining was performed on wild-type eye discs (Figures 3E–3E’). Endogenous SPARC staining colocalized with Vkg::GFP in the eye disc BM (Figures 3E’–3E’’). In contrast, eye disc-derived SPARC::HA localized in the eye disc posterior to the morphogenetic furrow but failed to colocalize with Vkg::GFP in the eye disc BM (Figures 3F’–3F’’). Furthermore, as observed with wing disc-derived SPARC, eye disc-derived SPARC::HA localized within fat body adipocyte borders, distinct from Col(IV) (Figure 3G’). This experiment was repeated using *Dll*-Gal4, which drives expression in the distal compartment of leg discs (Figures 3H–3J). Similar to the wing and eye discs, endogenous SPARC staining colocalized with Vkg::GFP in the leg disc BM (Figures 3H’–3H’’) whereas leg disc-derived SPARC::HA did not localize to the leg disc BM (Figures 3I’–3I’’). SPARC::HA localized to the distal compartment of the leg discs but failed to colocalize with Vkg::GFP in the leg disc BM (Figure 3I’). Moreover, as observed with wing disc- and eye disc-derived SPARC, leg disc-derived SPARC::HA did not localize to the fat body BM. The merged images between Vkg::GFP and anti-HA staining show SPARC::HA localized within fat body adipocyte borders, distinct from the localization of Col(IV) (Figure 3J’). Findings were consistent across all three leg disc types (Figure S3). Collectively, these data indicate epithelial disc-derived SPARC does not colocalize with Col(IV) at proximal or distal BMs. Rather, epithelial disc-derived SPARC diffuses to and associates at fat body adipocyte cell borders, distinct from Col(IV).

To confirm the specificity of the HA antibody to detect epithelial disc-derived SPARC::HA, SPARC::HA expression was knocked down in the wing, eye, or leg discs. SPARC::HA and *SPARC*^*RNAi*^ were simultaneously expressed in the imaginal discs using their respective Gal4 driver lines (*en*-Gal4, *pGMR*-Gal4, *Dll*-Gal4). Compared to tissues expressing SPARC::HA, tissues co-expressing SPARC::HA and *SPARC*^*RNAi*^ showed a marked decrease in the staining intensity of SPARC::HA in their respective compartments with no apparent defects in Col(IV) distribution (Figures 3K–3M and S4). Furthermore, the simultaneous expression of SPARC::HA and *SPARC*^*RNAi*^ in the wing discs using *en*-Gal4 resulted in fewer immunoreactive punctae in the fat body (Figures 3L–3L’). Western blot analyses confirmed SPARC::HA was knocked down in the imaginal discs (Figure 3M). Collectively, these data demonstrate that despite being able to diffuse to the fat body, epithelial disc-derived SPARC fails to colocalize with Col(IV) in proximal or distal BMs, suggesting functions other than a Col(IV) chaperone-like activity.

### Exogenous co-expression of SPARC and Col(IV) in the wing, leg, and eye discs does not result in their localization to the BM encapsulating the discs

**Figure 4:**
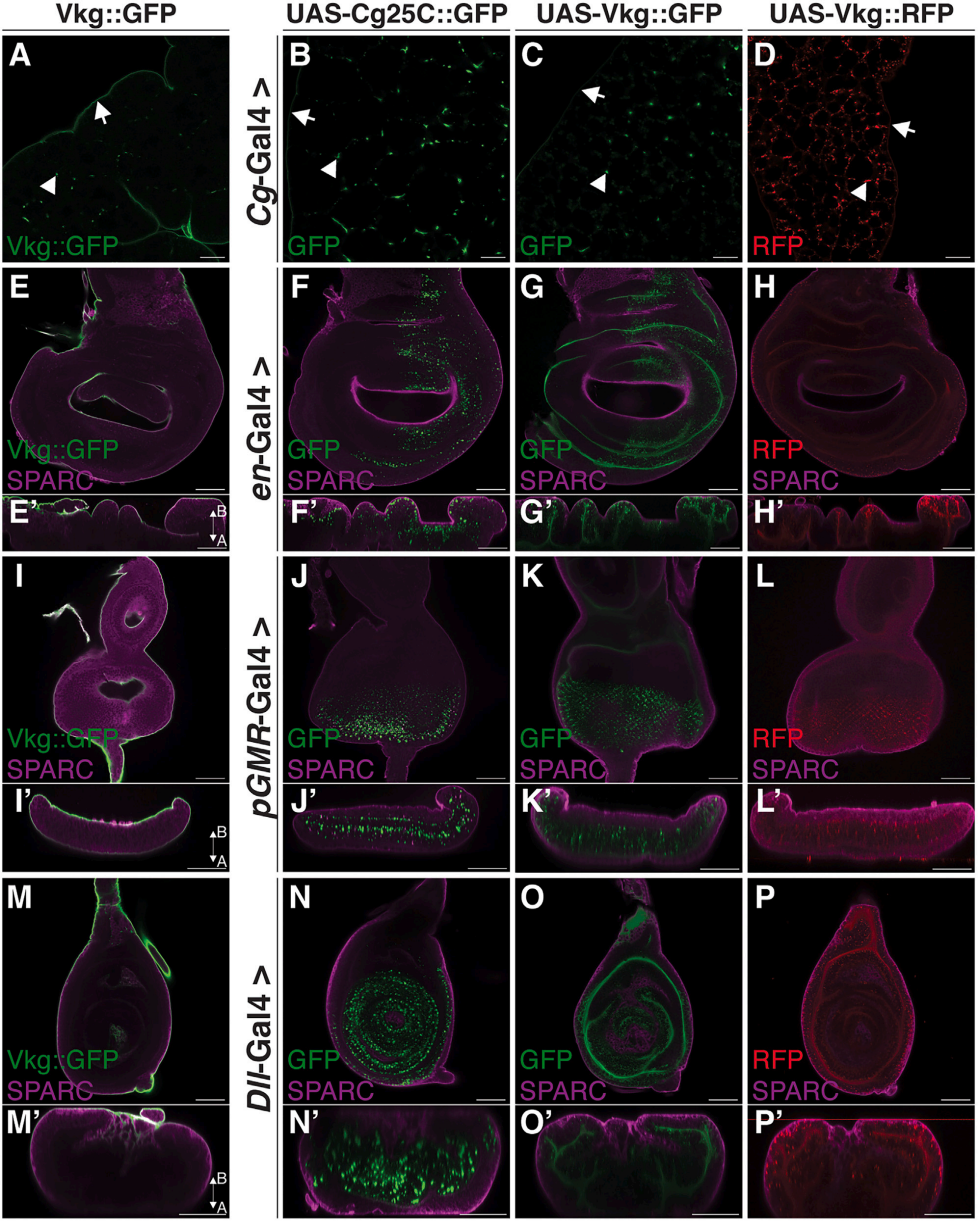
Fluorescently-tagged UAS-Col(IV) α-chains do not localize to the wing, eye, or leg disc BM (A) Representative image of a wild-type fat body expressing endogenous Vkg::GFP to visualize Vkg in the fat body BM. White arrowheads indicate Vkg::GFP signal in CIVICs. Arrows indicate the Vkg::GFP signal in the BM encapsulating the fat body. (B–D) Immunostaining of fat body-derived UAS-Cg25C::GFP (B), UAS-Vkg::GFP (C), and UAS-Vkg::RFP (D) expressed under control of the *Cg*-Gal4 promoter. White arrowheads indicate Vkg::GFP signal in CIVICs. Arrows indicate the Vkg::GFP signal in the BM encapsulating the fat body. (E) Immunostaining of endogenous SPARC in a wing disc extracted from a wild-type larvae expressing endogenous SPARC and Vkg. Vkg::GFP to visualize Vkg in the wing disc BM. (F–H) Immunostaining of endogenous SPARC (magenta) and wing disc-derived UAS-Cg25C::GFP (F), UAS-Vkg::GFP (G), and UAS-Vkg::RFP (H) expressed under control of the *en*-Gal4 promoter. Cross-sections of wing discs show the localization of individual UAS-Col(IV) chains expressed by the wing discs (F’–H’). (I) Immunostaining of endogenous SPARC in an eye disc extracted from a wild-type larvae expressing endogenous SPARC and Vkg. Vkg::GFP to visualize Vkg in the eye discs BM. (J–L) Immunostaining of endogenous SPARC (magenta) and eye disc-derived UAS-Cg25C::GFP (I), UAS-Vkg::GFP (J), and UAS-Vkg::RFP (K) expressed under control of the *pGMR*-Gal4 promoter. Cross-sections of eye discs show the localization of individual UAS-Col(IV) chains expressed by the eye discs (J′–L′). (M) Immunostaining of endogenous SPARC in a leg disc extracted from a wild-type larvae expressing endogenous SPARC and Vkg. Vkg::GFP to visualize Vkg in the leg discs BM. (N–P) Immunostaining of endogenous SPARC (magenta) and leg disc-derived UAS-Cg25C::GFP (N), UAS-Vkg::GFP (O), and UAS-Vkg::RFP (P) expressed under control of the *Dll*-Gal4 promoter. Cross-sections of leg discs show the localization of individual UAS-Col(IV) chains expressed by the leg discs (N’–P’). Apical and basal orientation is provided in panels (E’, I’, and M’). Scale bars represent 25 μm. Data shown are representative of a minimum of three biological replicates. See also Figure S5.

A caveat of the approach used to show epithelial disc-derived SPARC does not colocalize with Col(IV) in BMs is that SPARC and Col(IV) may not be produced by the same cells in these tissues. To address this possibility, we sought to co-express SPARC and UAS-Col(IV) α-chains in the wing, eye, and leg discs to determine if this resulted in the colocalization of SPARC and Col(IV). We first confirmed that the fluorescently-tagged UAS-Col(IV) α-chains (UAS-Cg25C::GFP, UAS-Vkg::GFP, and UAS-Vkg::RFP) phenocopy the distribution of endogenous Col(IV) in proximal and distal sites when expressed in the fat body (Figure S5). We then determined if exogenous expression of Col(IV) in the wing discs could contribute to the formation of the wing disc BM. The expression of UAS-Cg25C::GFP, UAS-Vkg::GFP, or UAS-Vkg::RFP in the wing discs failed to integrate into the wing disc BM (Figures 4B–4D). Rather, wing disc-derived UAS-Cg25C::GFP localized to folds in the wing discs, with a predominance of large intracellular punctae (Figure 4B). Wing disc-derived UAS-Vkg::GFP and UAS-Vkg::RFP also localized to folds in the wing discs. However, fewer intracellular Col(IV) punctae were observed compared to UAS-Cg25C::GFP (Figures 4C and 4D). Moreover, none of the fluorescently-tagged UAS-Col(IV) α-chain constructs localized to the fat body BM (data not shown). Collectively, these data indicate the expression of individual UAS-Col(IV) α-chain constructs does not result in the localization of Col(IV) in the wing disc BM.

In *Drosophila*, Col(IV) protomers are composed of two α1 chains and one α2 chain. The wing discs do not express endogenous Col(IV), and thus are not capable of forming protomers when only one of the fluorescently-tagged UAS-Col(IV) α-chains is expressed. Similar to the fat body, the eye and leg discs express Col(IV) and thus are expected to produce protomers that integrate the fluorescently-tagged UAS-Col(IV) α-chains. We therefore examined the distribution of the fluorescently-tagged UAS-Col(IV) α-chains when expressed by the eye or leg discs, using endogenous SPARC as a marker for the BM. Endogenous Vkg::GFP was used as a control to show the localization of endogenous SPARC and Col(IV) in the BM of eye and leg discs (Figures 4I and 4M). UAS-Cg25C::GFP, UAS-Vkg::GFP, or UAS-Vkg::RFP expressed by the eye or leg discs did not colocalize with SPARC in the BM of the eye discs (Figures 4J–4L, respectively). Rather, eye or leg disc-derived UAS-Cg25C::GFP localized predominantly in large intracellular punctae in the eye discs posterior to the morphogenetic furrow or leg discs distal compartment (Figures 4J–4L, respectively). SPARC staining mainly localized to the BM of the eye or leg discs, with a subset of SPARC colocalizing with Col(IV) punctae (Figures 4J–4L, respectively). Furthermore, UAS-Vkg::GFP and UAS-Vkg::RFP expressed in the eye discs localized to the ommatidia BM, as previously reported.^27^ The localization pattern of eye or leg disc-derived UAS-Cg25C::GFP, UAS-Vkg::GFP, and UAS-Vkg::RFP shares a level of similarity to their counterparts being expressed in the wing discs. Thus, the expression of only one fluorescently-tagged UAS-Col(IV) α-chain in the imaginal discs does not yield a protein product that associates with the BM encapsulating the discs, despite the endogenous expression of the α1 and α2 chains of Col(IV).

**Figure 5:**
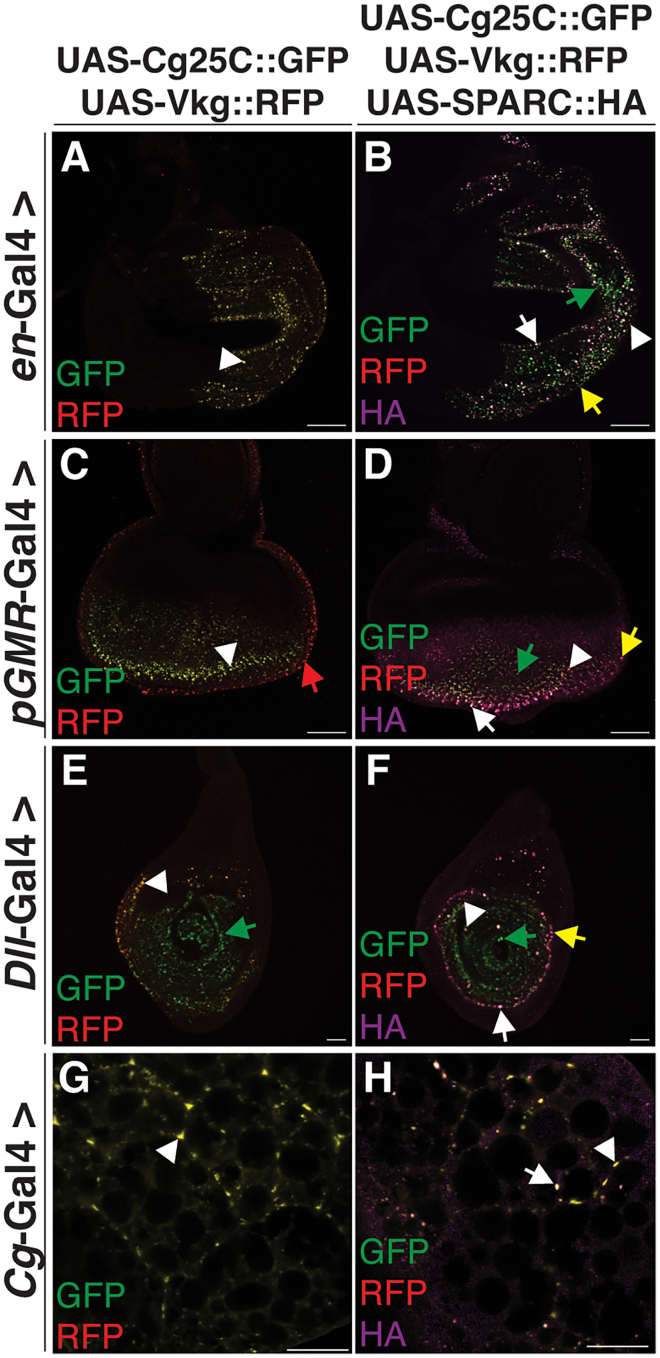
Co-expression of fluorescently-tagged UAS-Col(IV) α-chains with or without UAS-SPARC::HA do not localize to the wing, eye, or leg disc BM (A and B) Immunostaining of wing disc-derived UAS-Cg25C::GFP and UAS-Vkg::RFP (A), or UAS-Cg25C::GFP, UAS-Vkg::RFP, and UAS-SPARC::HA (magenta) (B) expressed under control of the *en*-Gal4 promoter. (C and D) Immunostaining of eye disc-derived UAS-Cg25C::GFP and UAS-Vkg::RFP (C), or UAS-Cg25C::GFP, UAS-Vkg::RFP, and UAS-SPARC::HA (magenta) (D) expressed under control of the *pGMR*-Gal4 promoter. (E and F) Immunostaining of leg disc-derived UAS-Cg25C::GFP and UAS-Vkg::RFP (E), or UAS-Cg25C::GFP, UAS-Vkg::RFP, and UAS-SPARC::HA (magenta) (F) expressed under control of the *Dll*-Gal4 promoter. (G and H) Immunostaining of fat body-derived UAS-Cg25C::GFP and UAS-Vkg::RFP (G), or UAS-Cg25C::GFP, UAS-Vkg::RFP, and UAS-SPARC::HA (magenta) (H) expressed under control of the *Cg*-Gal4 promoter. White arrowheads indicate the colocalization of UAS-Cg25C::GFP and UAS-Vkg::RFP. Green arrows indicate the localization of UAS-Cg25C::GFP only positive punctae. Red arrows indicate the localization of UAS-Vkg::RFP only positive punctae. Yellow arrows indicate the localization of UAS-SPARC::HA only positive punctae. White arrows indicate the colocalization of UAS-Cg25C::GFP, UAS-Vkg::RFP, and UAS-SPARC::HA. Scale bars represent 25 μm. Data shown are representative of a minimum of three biological replicates.

We next set out to determine if driving expression of both fluorescently-tagged α1 and α2 chains results in a Col(IV) heterotrimer that is integrated into the BM encapsulating the discs. Co-expression of UAS-Cg25C::GFP and UAS-Vkg::RFP in the wing, eye, or leg discs resulted in the intracellular punctate localization of UAS-Cg25C::GFP and UAS-Vkg::RFP, with no integration into the BM. Not surprisingly, a subset of punctae exhibited only RFP or GFP fluorescence (Figures 5A, 5C, and 5E).

Lastly, to test if SPARC and Col(IV) co-secretion enables their integration into BMs, we co-expressed UAS-Cg25C::GFP and UAS-Vkg::RFP with SPARC::HA. Interestingly, their co-expression in the wing, eye, or leg discs failed to result in their integration into the BM encapsulating the discs. Rather, UAS-Cg25C::GFP, UAS-Vkg::RFP, and SPARC::HA localized as intracellular punctae, with punctae showing differing degrees of colocalization (Figures 5A, 5C, and 5E, Video S1).

Video S1. This video shows a 3D reconstruction of third instar wing discs expressing UAS-Cg25C::GFP (green), UAS-Vkg::RFP (red) and UAS-SPARC::HA (magenta) and the distribution of punctae throughout the tissue

Given these unexpected results, we further validated our experimental approach by co-expressing UAS-Cg25C::GFP and UAS-Vkg::RFP with or without SPARC::HA in the larval fat body. UAS-Cg25C::GFP and UAS-Vkg::RFP colocalized in the BM of the fat body and CIVICs in the absence and presence of SPARC::HA (Figures 5G and 5H), confirming our approach. Collectively, these data demonstrate the co-secretion of SPARC and Col(IV) by epithelial cells in the wing, eye, and leg discs does not enable their colocalization in BMs. Thus, SPARC produced by these tissues does not function as a Col(IV) chaperone-like protein. Moreover, these results show that Col(IV) produced by the wing, eye, and leg discs does not contribute to the formation of the BM encapsulating these discs, suggesting a different, albeit important, unknown function for this collagen.

### Deletion of SPARC domains does not enable wing disc-derived SPARC to associate with Col(IV) in BMs

**Figure 6:**
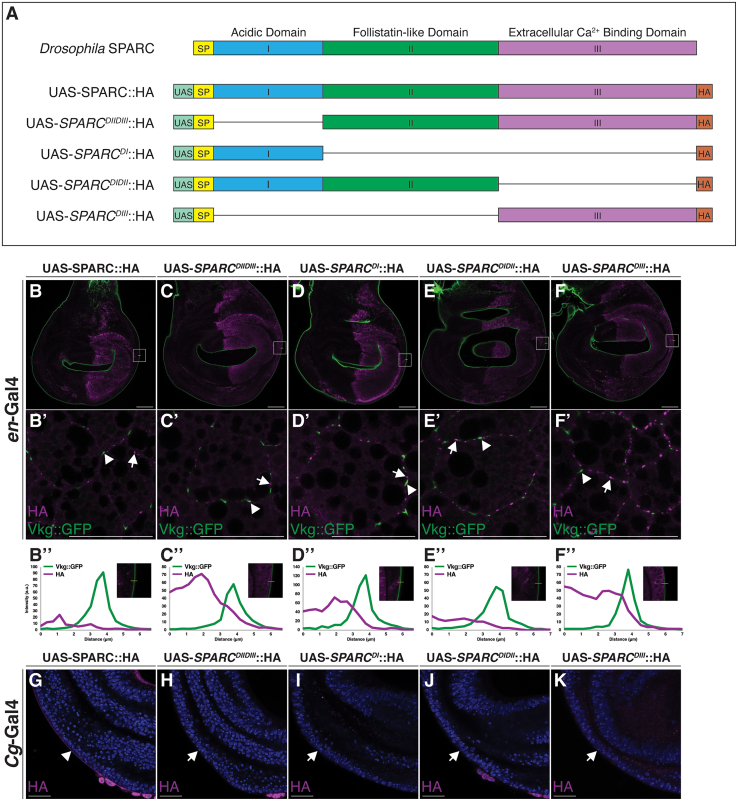
Full-length SPARC derived from the fat body is required for BM localization to the wing disc BM (A) Schematic depiction of full-length SPARC and the additional transgenic UAS-SPARC::HA modular constructs generated using gateway cloning technology. The schematic indicates the UAS promoter (UAS; teal), a signal peptide (SP; yellow), SPARC domain I (blue), SPARC domain II (green), SPARC domain III (purple), and C-terminal HA tag (orange). (B–F) Immunostaining of wing discs (B–F) and fat bodies (B’–F’) from larvae expressing the indicated UAS-SPARC::HA modular constructs in the wing discs under control of *en*-Gal4. Wing discs and fat bodies were stained for the UAS-SPARC::HA modular constructs using HA (magenta) and Vkg::GFP (green) was used to visualize Vkg in the wing disc (B–F) and fat body (B’–F’) BM. Arrowheads indicate Vkg::GFP signal. Arrows indicate UAS-SPARC::HA modular construct staining that does not colocalize with Vkg::GFP. Intensity plot profiles for Vkg::GFP (green) and HA (magenta) along the yellow solid line (B”–F”). Cutout in each plot shows the magnified image and region of the discs from which the intensity plots were generated. (G–K) Immunostaining of wing discs (G–K) from larvae expressing the indicated UAS-SPARC::HA modular constructs in the fat body under control of the *Cg*-Gal4 promoter. Wing discs were stained for SPARC constructs using HA (magenta) and Vkg::GFP (green) was used to visualize Vkg in the wing disc BM. Nuclei were stained using DAPI (blue). Arrowheads indicate UAS-SPARC::HA staining in the BM of the wing discs. Arrows indicate the absence of UAS-SPARC::HA modular construct staining that does not localize to the wing disc BM. Scale bars represent 25 μm. Data shown are representative of a minimum of three biological replicates. See also Figure S6.

To test if a domain of SPARC functions to prevent the localization of wing disc-derived SPARC to fat body adipocyte cell borders, we generated transgenic flies capable of expressing HA-tagged full-length SPARC and SPARC with various domain deletions (Figures 6A and S6). All the UAS-SPARC::HA domain variants retained the signal peptide of full-length SPARC to ensure proper secretion and were expressed in the wing discs using *en*-Gal4. Despite the presence of Col(IV) in the wing disc BM, none of the SPARC::HA domain variants or full-length SPARC::HA localized to the wing disc BM (Figures 6B–6F and 6B”–6F”). In the fat body, wing disc-derived SPARC::HA domain variants and full-length SPARC exhibited the same tissue distribution wherein HA staining was restricted to the adipocyte cell border in a punctate manner, distinct from Col(IV) (Figures 6B’–6F’). While surprising, these data suggest that the inability of wing disc-derived SPARC to colocalize with Col(IV) is not mediated by a single domain of SPARC.

### Domain I of SPARC is essential for the association of fat body-derived SPARC with Col(IV) at distal sites

Given that none of the SPARC::HA domain variants prevented the Col(IV)-independent localization of wing-disc derived SPARC, we next determined the roles of the various domains of SPARC in the colocalization of fat body-derived SPARC with Col(IV) in the wing discs. We used the same UAS-SPARC::HA domain variants as in the previous experiment, but expressed under control of the *Cg*-Gal4 promoter. We examined the distribution of fat body-derived SPARC::HA domains variants in the wing disc BM. As expected, full-length SPARC::HA localized to the wing disc BM (Figure 6G). In contrast, none of the SPARC::HA domain variants, including *SPARC*^*DIIDIII*^::HA, localized to the wing disc BM (Figures 6H–6K), suggesting a role for domain I of SPARC in maintaining its association with Col(IV) beyond the fat body.

### Domain II and III of SPARC are necessary and sufficient for the diffusion of fat body-derived Col(IV) but not SPARC to distal sites and rescues larval lethality

**Figure 7:**
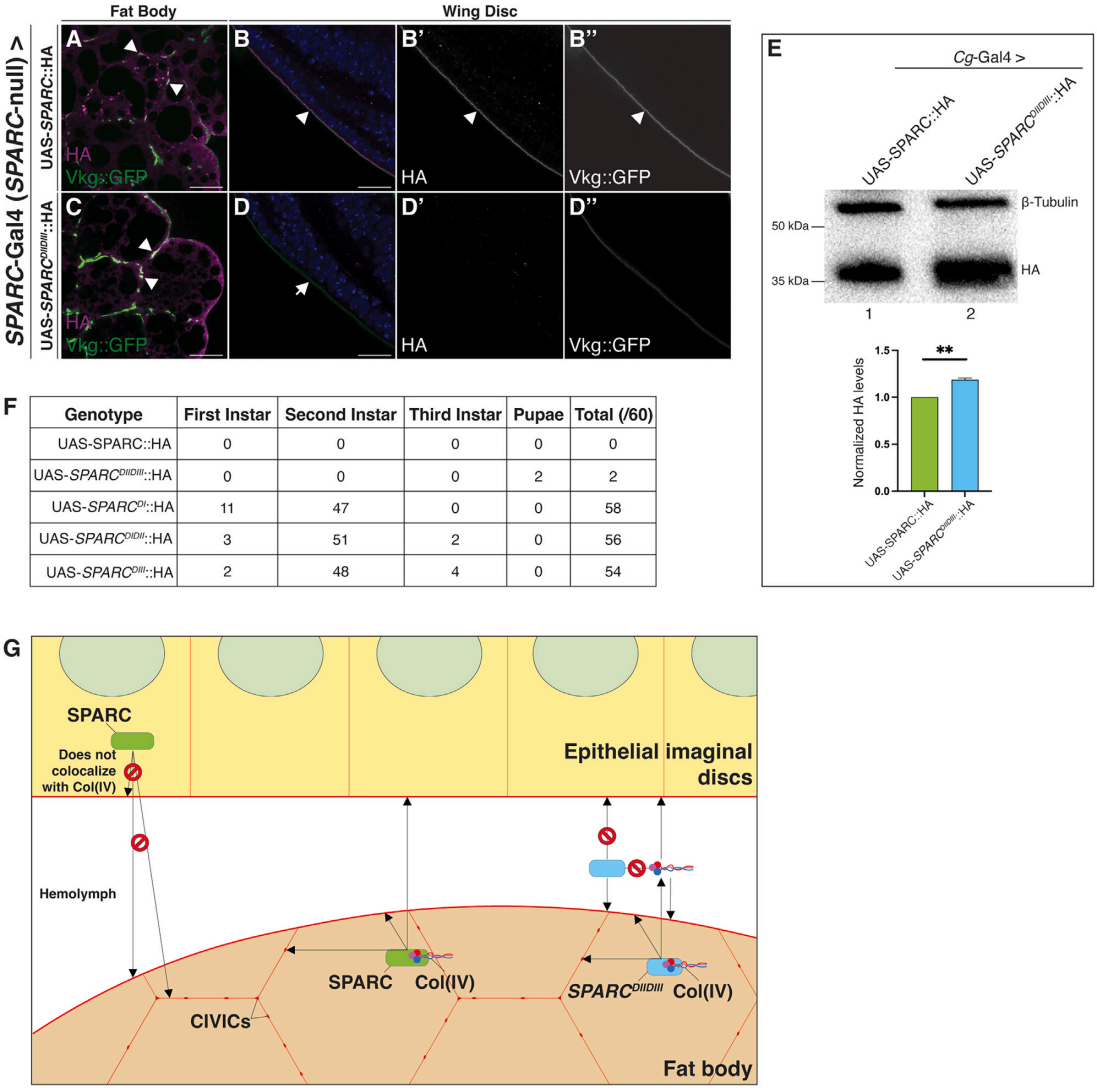
Domain I of SPARC is required for the diffusion of fat body-derived SPARC to the wing disc BM (A–D”) Immunostaining of larval fat bodies and wing discs expressing SPARC::HA (A-B″) and *SPARC*^*DIIDIII*^::HA (C–D”) in a *SPARC*-null background. Arrowheads indicate overlap between HA (magenta) and Vkg::GFP (green) at CIVICs, the surface of the fat body BM, and the wing disc BM. Arrows indicate the absence of HA staining in the wing disc BM. Scale bars represent 25 μm. Data shown are representative of a minimum of three biological replicates. (E) Western blot showing levels of SPARC::HA (lane 1) and *SPARC*^*DIIDIII*^::HA (lane 2) in third instar larval hemolymph. β-tubulin (∼55 kDa) was used as a loading control. Data were calculated as mean ΗΑ intensity normalized to β-tubulin and are represented as the mean ± SEM of three biological replicates. Statistical significance was assessed by a one-sample t-test t_(df=2)_ = 10.06, ∗∗*p* = 0.0097. (F) Larval lethality assay scoring the number of dead progeny at each stage of development for the various transgenic UAS-SPARC::HA modular constructs when expressed in a *SPARC*-null background. A total of 60 progeny (three biological replicates, *n* = 20 per replicate) were scored for each genotype. (G) Model depicting the tissue-specific differences in the ability for SPARC to colocalize with Col(IV). Epithelial disc-derived SPARC is unable to colocalize with Col(IV) in the epithelial discs or fat body BM. Fat body-derived SPARC colocalizes with Col(IV) in both the fat body and epithelial discs BM. In contrast, *SPARC*^*DIIDIII*^::HA colocalizes with Col(IV) in the fat body but is unable to diffuse to and colocalize with Col(IV) in the wing disc BM.

To test the possibility that domain I of SPARC is required for the diffusion of fat body-derived SPARC to the wing disc BM, we compared the distribution of SPARC::HA to *SPARC*^*DIIDIII*^::HA expressed under the control of the endogenous *SPARC*-Gal4 promoter in a *SPARC*-null background. Immunostaining of SPARC::HA indicated colocalization with Col(IV) in both the fat body and wing discs, as expected (Figures 7A–7B”). In contrast, immunostaining of *SPARC*^*DIIDIII*^::HA was present only in the fat body and absent in the wing discs (Figures 7C–7D”). However, Col(IV) was present in the wing disc BM, indicating that domain I of SPARC is essential for the diffusion of SPARC to the wing discs from the fat body. Interestingly, levels of *SPARC*^*DIIDIII*^::HA in the larval hemolymph were higher than SPARC::HA (Figure 7E), suggesting a possible accumulation. The expression of *SPARC*^*DIIDIII*^::HA in a *SPARC*-null background rescued to adulthood the second instar larval lethality associated with the loss of SPARC (Figure 7F). None of the other SPARC domain variants other than full-length SPARC::HA rescued larval lethality (Figure 7F). Thus, these data indicate the chaperone-like activity of SPARC does not need to be maintained beyond the fat body for Col(IV) diffusion and polymerization at distal sites, which is required for survival.

## Discussion

Our previous work and that of others has established an essential role of fat body-derived SPARC for the diffusion and integration of Col(IV) into BMs at distal sites, thus serving a chaperone-like function for Col(IV). In contrast to fat body-derived SPARC, wing disc-derived SPARC does not localize to the wing disc BM nor does it colocalize with Col(IV) in fat body adipocyte BMs. Rather, wing disc-derived SPARC localizes to fat body adipocyte cell borders in a punctate manner distinct from Col(IV), suggesting a distinct functional role.^11^ In the present study, we explored possible explanations for the inability of wing disc-derived SPARC to colocalize with Col(IV). We excluded the possibility that this is simply due to an inability of wing disc-derived SPARC to displace fat body-derived SPARC from Col(IV). We show by enzymatic digestion that the N-glycosylation patterns of fat body- and wing disc-derived SPARC are similar and that removal of N-glycosylation did not alter their ability to colocalize with Col(IV). Moreover, co-expressing SPARC and Col(IV) within the same cells of the wing, eye, and leg discs did not enable the colocalization of SPARC and Col(IV) in their respective BMs nor in the fat body BM. These data indicate fat body- and wing disc-derived SPARC are functionally distinct from one another and that the inability of wing disc-derived SPARC from colocalizing with Col(IV) is not explained by structural differences in SPARC synthesized from these tissues.

The current model for the chaperone-like function of fat body-derived SPARC holds that SPARC associates with Col(IV) intracellularly to prevent its precocious polymerization and that upon secretion, continues to prevent aberrant Col(IV) polymerization until it reaches its destination at proximal or distal sites. Our SPARC domain deletion variants indicate domain II and III are sufficient for the chaperone-like function of fat body-derived SPARC, allowing Col(IV) to both form BMs at the fat body and diffuse to and integrate into BMs at distal sites. However, these studies show that SPARC does not need to accompany Col(IV) to distal sites for BM assembly. Importantly, the expression of SPARC domains II and III in a *SPARC*-null background was sufficient to rescue to adulthood the larval lethality associated with the loss of SPARC and enabled Col(IV) diffusion to distal BMs. This indicates the Col(IV) chaperone-like function of SPARC is essential and required only at the fat body to ensure Col(IV) protomers are secreted and delivered to the hemolymph.

The finding that removing the N-glycosylation acceptor site of *Drosophila* SPARC did not promote the colocalization of wing disc-derived SPARC with Col(IV) was unexpected given that removal of N-glycosylation in mammalian platelet-derived SPARC increases its collagen-binding affinity to levels comparable to bone-derived SPARC. Our studies further show that removal of N-glycosylation does not impair the ability for fat body-derived SPARC to colocalize with Col(IV) in CIVICs and in the fat body BM, in addition to diffusing to distal sites. Thus, unlike in mammals, glycosylation does not appear to play a determining role in the ability of *Drosophila* SPARC to associate with Col(IV). Although our enzymatic digestion studies indicate the N-glycosylation patterns of fat body- and wing disc-derived SPARC show no overt differences, it remains possible that subtle differences in N-glycosylation branching or fucosylation may be present.^28^

Previous work by our laboratory reported the absence of CIVICs in fat bodies of second instar larvae using a Col(IV) antibody;^11^ however, in this study we report the presence of these structures at this stage. CIVICs are distinct from BMs, thicker in section, and mediate inter-adipocyte adhesion in *Drosophila*. Since our previous study, we adapted the use of a GFP protein trap inserted into the *Drosophila vkg* gene, which produces a functional GFP-tagged version of Vkg (Vkg::GFP) that enables enhanced visualization of Col(IV). Our current study revealed Vkg::GFP signal distinct from BMs in second instar larval fat bodies, thus demonstrating CIVICs are present in second instar larval fat bodies and may be essential for adipocyte cell-cell adhesion at this stage.

The inability of wing disc-derived SPARC to colocalize with Col(IV) in its own BM and the BM of the fat body is also observed with eye and leg disc-derived SPARC. We previously determined SPARC and Col(IV) first colocalize in the trans-Golgi with little or no colocalization within the ER or cis-Golgi,^11^ raising the possibility that SPARC and Col(IV) need to associate intracellularly and be co-secreted to colocalize in BMs. Overexpression of Col(IV) using fluorescently-tagged UAS-Col(IV) constructs indicated expression of a single Col(IV) α-chain is not sufficient to localize to the BM encapsulating the wing, eye, or leg discs. In the eye discs, expression of a single UAS-Col(IV) α-chain localized with the ommatidial BM, consistent with a recent report.^27^ Surprisingly, co-expression of both UAS-Col(IV) α-chains in the absence or presence of SPARC::HA resulted in their colocalization in punctae and failed to result in the integration of Col(IV) in the BM encapsulating the discs. Moreover, the distribution of punctae varied between the wing, eye, and leg discs. The formation of Col(IV) trimers requires a complex machinery for several post-translational modifications, including prolyl hydroxylation, lysyl hydroxylation, and glycosylation of hydroxylysine residues.^8^^,^^12^^,^^29^^,^^30^^,^^31^ It is possible that exogenous expression of Col(IV) trimers may lead to Col(IV) misfolding as a result of the absence of such enzymatic modifications in these tissues. A failure to form proper Col(IV) trimers could thereby prevent Col(IV) from interacting with SPARC. Another possibility is that the function(s) of imaginal disc-derived SPARC may be independent of BM formation and maintenance. For example, studies in *Drosophila* indicate wing disc-derived SPARC may serve a protective role in the wing disc cells undergoing cell competition.^32^

In the current study, we used *en*-Gal4 to drive expression of UAS constructs in the posterior compartment of the wing discs and make conclusions regarding wing disc-derived SPARC. It is, however, important to note that *en*-Gal4 is also expressed in other tissues, including the posterior compartments of all imaginal discs and the larval epidermis.^33^^,^^34^ Moreover, a limitation of this study is the use of a single binary (UAS/Gal4) system to spatiotemporally express multiple UAS constructs in the same organism. The use of two binary systems (such as UAS/Gal4 and LexA/Aop) could provide further evidence to support our findings.

Fat body adipocytes are the principal source of BM components during *Drosophila* larval development.^8^^,^^35^ While the wing discs do not express Col(IV) and are fully reliant on the fat body, some tissues rely heavily on the integration of Col(IV) produced by the fat body into their BMs, although they contain cells that express Col(IV). Examples of such tissues include the ovary and eye discs, where stalk cells and eye disc ommatidia, respectively, have been shown to produce and integrate Col(IV) into their BMs. However, the BMs encapsulating these tissues were shown to be derived from the fat body.^27^^,^^36^ Moreover, the short-term loss of SPARC had no impact on BM maintenance but is required for repair following damage to the midgut.^37^ This raises the possibility that Col(IV) produced by these tissues serves BM-independent functions that may or may not involve SPARC.

Mimetic peptide analyses with mammalian SPARC have identified various functions associated with the three domains.^38^^,^^39^^,^^40^ Expression of UAS-SPARC::HA domain deletion constructs in the wing discs phenocopied the distribution of full-length SPARC::HA, suggesting the domain(s) responsible for the fat body Col(IV)-independent localization of wing disc-derived SPARC may be functionally redundant or bind to different ligands and/or receptors at adipocyte cell borders. This functional redundancy suggests wing disc-derived SPARC serves an important role in the fat body. Further studies are thus warranted to explore these functions.

When expressed by the fat body, only full-length SPARC::HA diffuses to and integrates into the BM of wing discs. Our studies indicate this diffusion requires the presence of domain I. This domain contains multiple low-affinity Ca^2+^-binding sites. It is well established that high-affinity Ca^2+^-binding to the EF-hands of SPARC domain III enhances its ability to bind collagens.^39^ However, there is no evidence to date indicating Ca^2+^-binding to domain I of SPARC impacts its ability to bind collagens. The hemolymph Ca^2+^ concentration is approximately 0.5 mM,^41^ which falls within the range of the dissociation binding constant (K_D_) of SPARC domain I for Ca^2+^ (10 μM–1 mM).^14^ Thus, it is anticipated that Ca^2+^-binding to and release from domain I of SPARC will be in a dynamic equilibrium state in the extracellular space. In the current study, we showed that removal of domain I of SPARC prevents SPARC from localizing to distal BMs. Moreover, western blot analyses of hemolymph revealed an increase in the amount of fat body-derived *SPARC*^*DIIDIII*^::HA compared to full-length SPARC::HA. Collectively, these data provide *in vivo* evidence indicating domain I of SPARC is required to maintain the extracellular association between SPARC and Col(IV). Moreover, the loss of domain I appears to prevent the diffusion of SPARC itself beyond the hemolymph. This suggests the activities of SPARC outside its role in the larval fat body are not essential to further development and survival to the adult stage.

### Limitations of the study

As discussed, limitations inherent in this study include the use of a single binary system incorporating siRNA and expression constructs to address the possibility Col(IV) in the fat body BM may already be saturated by fat body derived SPARC. An alternative approach would be the use of two binary systems (such as UAS/Gal4 and LexA/Aop), one to express SPARC siRNA in the fat body and the second to express SPARC::HA in the imaginal discs. Although our enzymatic digestion studies clearly indicate the N-glycosylation patterns of fat body- and wing disc-derived SPARC show no overt differences and that N-glycosylation is not required for its colocalization with Col(IV), the exact N-linked glycan profiles of *Drosophila* SPARC remain to be determined. A failure to form proper Col(IV) trimers could prevent Col(IV) from interacting with SPARC in imaginal discs. The formation of Col(IV) trimers is a complex process involving prolyl hydroxylation, lysyl hydroxylation, and glycosylation of hydroxylysine residues.^8^^,^^12^^,^^29^^,^^30^^,^^31^ As a result, it is possible that exogenously expressed Col(IV) may have misfolded as a result of enzymatic deficiencies in these tissues. Alternatively, the function(s) of imaginal disc-derived SPARC may be independent of BM formation and maintenance and should be the subject of further queries.

## Resource availability

### Lead contact

Further information and requests for resources and reagents should be directed to and will be fulfilled by the lead contact Samuel Delage (samuel.delage@mail.utoronto.ca).

### Materials availability

All fly lines generated for this study are available from the lead contact upon request.

### Data and code availability

Data: All data reported in this paper will be shared by the lead contact upon request.

Code: This paper does not report original code.

Other items: Any additional information required to reanalyze the data reported in this paper is available from the lead contact upon request.

## Acknowledgments

We thank Dr. Stéphane Noselli (UAS-Cg25C::GFP, UAS-Vkg::GFP, UAS-Vkg::RFP), Dr. Eduardo Moreno (UAS-SPARC::HA), the Bloomington Stock Center, Drosophila Genomics Resources Center, and Vienna Drosophila Resource Center for providing fly strains. This work was supported by NSERC Discovery grant 498474 (to M.J.R.).

## Author contributions

Conceptualization, S.D. and M.J.R.; methodology, S.D. and M.J.R.; investigation, S.D., A.Z., and W.Y.; writing—original draft, S.D., T.J.B., and M.J.R.; writing—review and editing, S.D., T.J.B., and M.J.R.; visualization, S.D., T.J.B., and M.J.R.; supervision, S.D. and M.J.R.; funding acquisition, M.J.R.

## Declaration of interests

The authors declare no competing interests.

## STAR★Methods

### Key resources table

**Table:** 

REAGENT or RESOURCE	SOURCE	IDENTIFIER
**Antibodies**		

Rabbit anti-Drosophila SPARC	Martinek et al.^42^	N/A
Mouse anti-HA	Thermofisher Scientific	Catalog #26183; RRID: AB_2535792
Guinea pig anti-Drosophila Collagen IV	Shahab and Ringuette^43^	N/A
Mouse anti-β-tubulin	Developmental Studies Hybridoma Bank	E7; RRID: AB_528499
HRP-conjugated anti-Mouse IgG	Cell Signaling Technology	Catalog #7076; RRID: AB_330924
HRP-conjugated anti-Rabbit IgG	Cell Signaling Technology	Catalog #7074; RRID: AB_2099233
DAPI (4′,6-diamidino-2-phenylindole)	Cell Signaling Technology	Catalog #4083
Rhodamine Phalloidin	Thermofisher Scientific	Catalog #R415
Donkey anti-Mouse IgG (H + L) Highly Cross-Adsorbed secondary antibody, Alexa Fluor 488	Invitrogen	Catalog #A32766; RRID: AB_2866493
Donkey anti-Mouse IgG (H + L) Highly Cross-Adsorbed secondary antibody, Alexa Fluor 555	Invitrogen	Catalog #A31570; RRID: AB_2536180
Donkey anti-Mouse IgG (H + L) Highly Cross-Adsorbed secondary antibody, Alexa Fluor 647	Invitrogen	Catalog #A32787; RRID: AB_2762830
Donkey anti-Rabbit IgG (H + L) Highly Cross-Adsorbed secondary antibody, Alexa Fluor 488	Invitrogen	Catalog #A32790; RRID: AB_2762833
Donkey anti-Rabbit IgG (H + L) Highly Cross-Adsorbed secondary antibody, Alexa Fluor 555	Invitrogen	Catalog #A31572; RRID: AB_162543
Goat anti-Guinea pig IgG (H + L) Highly Cross-Adsorbed secondary antibody, Alexa Fluor 647	Invitrogen	Catalog #A21450; RRID: AB_2535867

**Chemicals, peptides, and recombinant proteins**		

ECL Plus chemiluminescence	Invitrogen	Catalog #WP20005
Phenylmethanesulfonyl fluoride (PMSF) protease inhibitor cocktail	BioShop	Catalog #PMS444.5
Halt™ Phosphatase Inhibitor Cocktail	Thermofisher Scientific	Catalog #78420
Vectashield	Vector Laboratories	Catalog #H-1000-10
PNGase F	New England Biolabs	Catalog #P0702
Endo H	New England Biolabs	Catalog #P0704
Precision Plus Protein Dual Color Standards	BioRad	Catalog #1610374
BLUelf Prestained Protein Ladder	FroggaBio	Catalog #PM008-0500

**Experimental models: Organisms/strains**		

D. melanogaster: w^1118^	Bloomington Drosophila Stock Center	3605
D. melanogaster: vkg^G454^::GFP	Drosophila Genomics Resources Center	110692
D. melanogaster: *tub*-Gal80ts	Bloomington Drosophila Stock Center	7108
D. melanogaster: *SPARC*-Gal4	Bloomington Drosophila Stock Center	77473
D. melanogaster: *SPARC*^*1510D*^	Duncan et al. (2020)	N/A
D. melanogaster: *Cg*-Gal4	Bloomington Drosophila Stock Center	7011
D. melanogaster: *en*-Gal4, UAS-RFP	Bloomington Drosophila Stock Center	30557
D. melanogaster: *en*-Gal4	Bloomington Drosophila Stock Center	30564
D. melanogaster: *pGMR*-Gal4	Bloomington Drosophila Stock Center	9146
D. melanogaster: *Dll*-Gal4	Bloomington Drosophila Stock Center	3038
D. melanogaster: UAS-SPARC RNAi	Vienna Drosophila Resource Center	16678
D. melanogaster: UAS-Cg25C::GFP	Gift from Dr. Stéphane Noselli	N/A
D. melanogaster: UAS-Vkg:GFP	Gift from Dr. Stéphane Noselli	N/A
D. melanogaster: UAS-Vkg:RFP	Gift from Dr. Stéphane Noselli	N/A
D. melanogaster: UAS-SPARC::HA	Gift from Dr. Eduardo Moreno	N/A
D. melanogaster: UAS-*SPARC*^*ΔNG*^::HA	This study	N/A
D. melanogaster: UAS-*SPARC*^*DIIDIII:*^:HA	This study	N/A
D. melanogaster: UAS-*SPARC*^*DI*^::HA	This study	N/A
D. melanogaster: UAS-*SPARC*^*DIDII*^::HA	This study	N/A
D. melanogaster: UAS-*SPARC*^*DIII*^::HA	This study	N/A
D. melanogaster: UAS-SPARC::HA, vkg^G454^::GFP	This study	N/A
D. melanogaster: UAS-*SPARC*^*ΔNG*^::HA, vkg^G454^::GFP	This study	N/A
D. melanogaster: UAS-*SPARC*^*DIIDIII:*^:HA, vkg^G454^::GFP	This study	N/A
D. melanogaster: UAS-*SPARC*^*DI*^::HA, vkg^G454^::GFP	This study	N/A
D. melanogaster: UAS-*SPARC*^*DIDII*^::HA, vkg^G454^::GFP	This study	N/A
D. melanogaster: UAS-*SPARC*^*DIII*^::HA, vkg^G454^::GFP	This study	N/A

**Software and algorithms**		

ImageJ-FIJI	ImageJ	https://imagej.nih.gov/ij/index.html
Leica LASX	Leica-microsystem	http://www.leica-microsystems.com/products/microscope-software/
ImageLab	Bio-Rad	https://www.bio-rad.com/it-it/product/image-lab-software
GraphPad Prism	GraphPad	http://www.graphpad.com/scientific-software/prism/
Adobe Illustrator	Adobe	https://www.adobe.com/products/illustrator.html
Adobe Photoshop	Adobe	https://www.adobe.com/products/photoshop.html
Microsoft Excel	Microsoft	https://www.microsoft.com/en/microsoft-365/word?market=af

### Experimental model and study participant details

All experimental strains used in this study are listed in the key resources table and included both male and female organisms. Standard fly husbandry techniques and genetic methodologies were used to assess the mutations and transgenic constructs. All crosses were carried out at 25°C in standard vials unless indicated otherwise. Rescue crosses were carried out in vials. Crosses generating progeny for larval lethality assays were carried out in cages with grape juice-supplemented agar plates.

### Method details

#### Generation of mutant SPARC transgenic fly lines

cDNA for mutant SPARC constructs were synthesized by ThermoFisher Scientific using GeneArt Strings technology and inserted into pENTR 2B Dual selection vector from ThermoFisher Scientific at a 3:1 (insert:destination) vector molar ratio. The resultant entry vectors containing the desired SPARC constructs were recombined with the pPWH destination vector containing a UAS promoter and a C-terminus 3xHA tag. Expression vectors were injected into w1118, yw embryos for integration into the second chromosome attp16 locus by BestGene Inc. (Chino Hills, California). Flies containing the construct of interest were selected for using the miniwhite y+ marker (orange eyes) and were crossed with a double-balancer line (Bl/Cyo; TM2/TM6B) to establish the line.

#### SPARC lethality assessment

To assess larval lethality, rescue analyses were performed by crossing respective SPARC mutant flies with *SPARC*-null mutant flies (*SPARC*^*1510D*^). Crosses were carried out in cages and fly embryos were laid on grape juice-supplemented agar plates. Twenty first instar larvae of the appropriate genotypes were reared on yeast-supplemented apple agar plates at 25°C until all larvae died, with three such plates being examined for each genotype. Dead larvae were removed from each plate once a day. The stage at which larval lethality occurred was scored based on the size of the larvae mouth hooks and shape of their spiracles.^44^ For each genotype examined, the number of dead larvae at each instar was pooled from each of the three plates and shown in a table format.

#### Isolation of second and third instar fat bodies

Second instar larvae were placed in a dissecting well containing PBS. Forceps were used to pinch open the cuticle at the posterior end of the larvae. The anterior end was then held in place with forceps and the contents of the larvae were expelled from the cuticle by sliding the forceps in an anterior to posterior direction. Fat bodies were separated from other tissues and prepared for subsequent analysis. Third instar larvae were placed in a dissecting well containing PBS. Forceps were used to pinch open the cuticle at the posterior end of the larvae. The larvae were then inverted, and fat bodies were separated from other tissues and prepared for subsequent analysis.

#### Isolation of third instar wing and leg imaginal discs

Third instar larvae were placed in a dissecting well containing PBS. Forceps were used to pinch open the cuticle at two-thirds its body length. The anterior end was then inverted. The fat body, salivary glands, gut, and remaining tissues were discarded. The complex containing the wing and leg imaginal discs was transferred to microcentrifuge tubes for preparation for immunofluorescence.^45^

#### Isolation of third instar eye imaginal discs

Third instar larvae were placed in a dissecting well containing PBS. One pair of forceps was used to grab the mouth hooks while the other pair was used to grab the larva at nearly 1/3 its body length. The pair of forceps holding the mouth hooks was held steady while the other pair quickly pulled away the rest of the body. The complex containing the mouth hooks and eye-antennal discs was transferred to microcentrifuge tubes for preparation for immunofluorescence.^45^

#### Immunofluorescence

Tissue samples were fixed in 4% formaldehyde in PBS for 45 min. Samples were then washed 3 times 20 min in PBSTx (PBS, 0.1% Triton X-100), blocked with 5% Donkey serum for 30 min, and then incubated with primary antibody in Donkey serum overnight at 4°C. Tissues were washed and blocked again in Donkey serum for 1 h. Host-specific Alexa Fluor-conjugated secondary antibodies were added to the solution and incubated for 2 h. Tissue samples were washed 3 times in PBSTx before mounting on a slide coated with VECTASHIELD mounting medium (Vector Laboratories, VECTH1000). All preparations were imaged by confocal microscopy (Leica TCS SP8 non-resonant microscope). Image analysis was performed using ImageJ software.^46^ Tissues from a minimum of three biological replicates, n=20 larvae were imaged for each genotype and/or condition.

#### Imaging and image analysis

Confocal microscopy images were acquired using a Leica SP8 microscope equipped with a Plan-Apo 40X oil CS2 objective (NA 1.30). For intensity plot profiles, images were imported into FIJI-ImageJ and the images were split into individual channels. The same region of interest was selected for each channel. The plot profile function was used to display the density profile plot of each channel. The values for each channel were saved as a csv file. Graphs displaying the intensity profiles were made using Microsoft Excel.

#### Western blot analyses

Tissues were homogenized with a pestle in microcentrifuge tubes in lysis buffer (100 mM tris 4% SDS pH 6.8). Protein concentration was measured using a Pierce BCA Protein Assay Kit (Pierce, Rockford, IL). 5–20 μg of each sample were diluted in 2X Laemmli buffer (100 mM Tris pH 6.8, 200 mM DTT, 20% glycerol, 0.01% Bromophenol blue and 4% SDS), boiled for 5 min, loaded into a 4–20% SDS polyacrylamide gel. Electrophoresed proteins were transferred to PVDF membranes. Membranes were blocked in 5% bovine serum albumin in PBST (PBS, containing 0.1% Tween 20) for 1 h at room temperature prior to incubation with their appropriate primary antibodies at 4°C overnight. Membranes were washed 3 times with PBST for 10 min. Membranes were then incubated with their appropriate peroxidase-conjugated secondary antibodies for 1 h at room temperature. Membranes were exposed to chemiluminescent reagents (Invitrogen, WP20005) and visualized using BioRad Molecular Imager Gel Doc XR + Imaging System. Precision Plus Protein Dual Color Standards (Bio-Rad) or BLUelf Prestained Protein Ladder (FroggaBio) were used as a molecular mass marker.

#### Western blotting of larval hemolymph

Ten larvae of each genotype were bled in 20 μL of PBS containing 0.1 mg/mL of phenylthiourea to avoid melanization and 1 mg/mL of antiprotease cocktail. 10 μL of sample per genotype were diluted in 2X Laemmli buffer (100 mM Tris pH 6.8, 200 mM DTT, 20% glycerol, 0.01% Bromophenol blue and 4% SDS), boiled for 5 min, and loaded into a 4–20% SDS polyacrylamide gel. Electrophoresed proteins were transferred to PVDF membranes. Membranes were blocked in 5% bovine serum albumin in PBST (PBS, containing 0.1% Tween 20) for 1 h at room temperature prior to incubation with a mouse anti-GFP and mouse anti-HA antibody. Membranes were exposed to chemiluminescent reagents (Invitrogen, WP20005) and visualized using BioRad Molecular Imager Gel Doc XR + Imaging System. Precision Plus Protein Dual Color Standards (Bio-Rad) were used as molecular weight markers.

### Quantification and statistical analysis

GraphPad Prism software was used for graphic representation and statistical analysis. Data were calculated as mean ΗΑ intensity normalized to β-tubulin and are represented as the mean ± SEM in the graph found in Figure 7E. For statistical comparisons of SPARC::HA and *SPARC*^*DIIDIII*^::HA in Figure 7E, a one-sample t-test was used (GraphPad Prism MacOS Version 10). Microsoft Excel was used for graphical representation of the intensity plot profiles in Fig ures 2, 3, and 6.

#### Temperature-sensitive knockdown of SPARC

Flies were reared in cages with grape juice-supplemented agar plates. Flies laid on the grape juice-supplemented agar plates for 4 hours to ensure consistent staging. Plates were then placed at 18°C to prevent *SPARC*-Gal4 expression. First instar larvae expressing Gal80^TS^, *SPARC*-Gal4, and UAS-*SPARC*^*RNAi*^ were isolated and transferred to 29°C to enable *SPARC*-Gal4 expression. The survival of the flies was assessed. Timepoint experiments were used to determine the effectiveness of the knockdown. First instar larvae were left at 29°C to develop for 0h, 12h, 18h and 24h. Western blot analyses were then performed on the larval cell lysates at these timepoints.
