# Supplementary material for: Drosophila SPARC collagen IV chaperone-like activity essential for development is unique to the fat body
Source: iScience. 2025 Feb 27;28(4):112111. doi: 10.1016/j.isci.2025.112111 (PMC12002606; doi:10.1016/j.isci.2025.112111)
Supplement: Document S1. Figure S1–S6 [file mmc1.pdf]

iScience, Volume *28*

Supplemental information

Drosophila SPARC collagen IV chaperone-like activity essential for development is unique to the fat body

Samuel Delage, Arya Zadhoosh, William You, Theodore Joseph Brown, and Maurice Joseph Ringuette

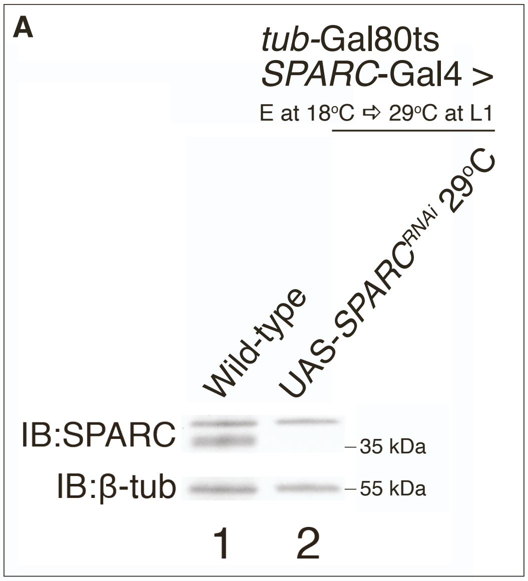

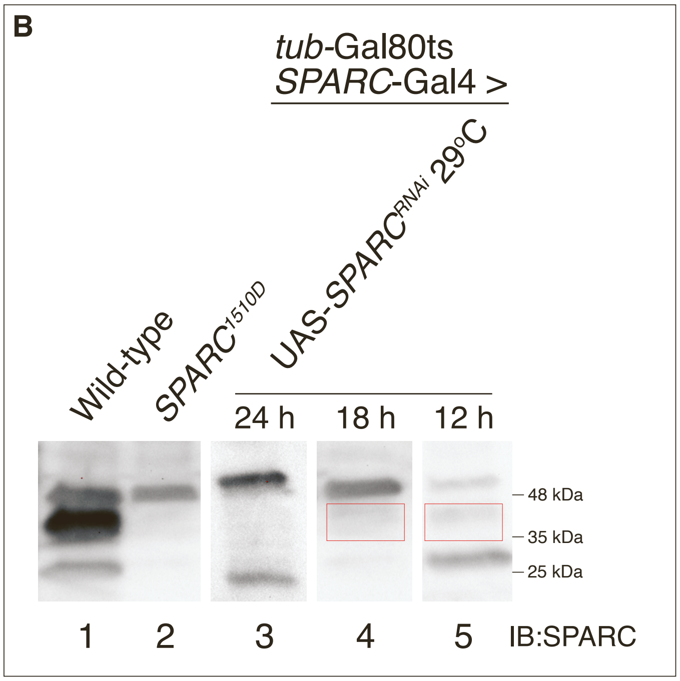

Figure S1. Time course of SPARC knockdown by RNAi following activation of *SPARC^{RNAi}*, related to Figure 1.

(A) Immunoblotting with anti-SPARC antibody. Western blot analysis using whole fly lysates prepared from wild-type flies (lane 1) and *SPARC^{RNAi}* flies expressing *SPARC*-Gal4 and tub-Gal80^{TS} transferred to 29°C following embryogenesis (lane 2).

(B) Western blot was analyzed by immunoblotting with anti-SPARC antibody. Western blot analysis using whole larvae lysates prepared from wild-type flies (lane 1), *SPARC*-null flies (*SPARC^{1510D}*, lane 2), and *SPARC^{RNAi}* flies transferred to 29°C following embryogenesis for 24 h (lane 3), 18 h (lane 4), and 12 h (lane 5). Lanes 3-5 are representative images from different blots run in parallel with lanes 1-2.

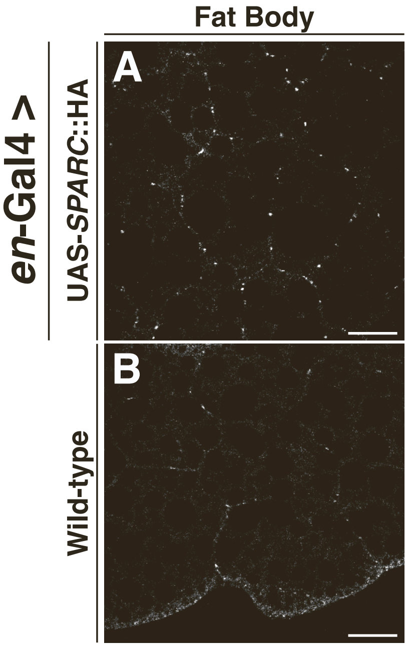

Figure S2. Verification of specificity for HA staining in larval fat bodies, related to Figure 1. (A-B) HA immunostaining of wing disc-derived SPARC::HA (A) and wild-type (B) larval fat bodies. Scale bars represent 25 μm. Data shown are representative of a minimum of three biological replicates.

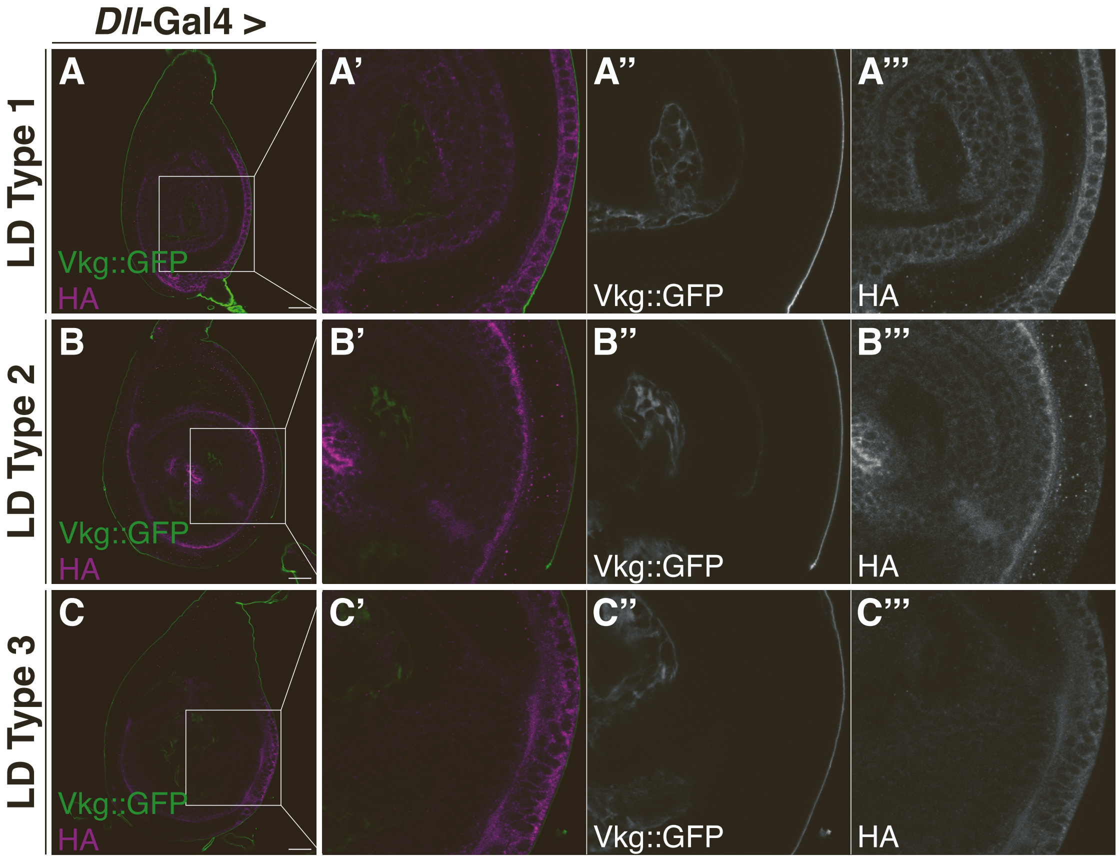

Figure S3: Leg disc-derived SPARC::HA does not colocalize with Col(IV) in any of the three leg disc types, related to Figure 3.

(A-A''') HA immunostaining of leg disc-derived SPARC::HA in leg disc type 1. HA immunostaining of third instar larval type 1 leg discs isolated from larvae expressing leg disc-derived SPARC::HA. Vkg::GFP (green) was used to visualize *Vkg*. Scale bars represent 25 µm.

(B-B''') HA immunostaining of leg disc-derived SPARC::HA in leg disc type 2. HA immunostaining of third instar larval type 2 leg discs isolated from larvae expressing leg disc-derived SPARC::HA. Vkg::GFP (green) was used to visualize *Vkg*. Scale bars represent 25 µm.

(C-C''') HA immunostaining of leg disc-derived SPARC::HA in leg disc type 3. HA immunostaining of third instar larval type 3 leg discs isolated from larvae expressing leg disc-derived SPARC::HA. Vkg::GFP (green) was used to visualize *Vkg*. Scale bars represent 25 µm.

Data shown are representative of a minimum of three biological replicates.

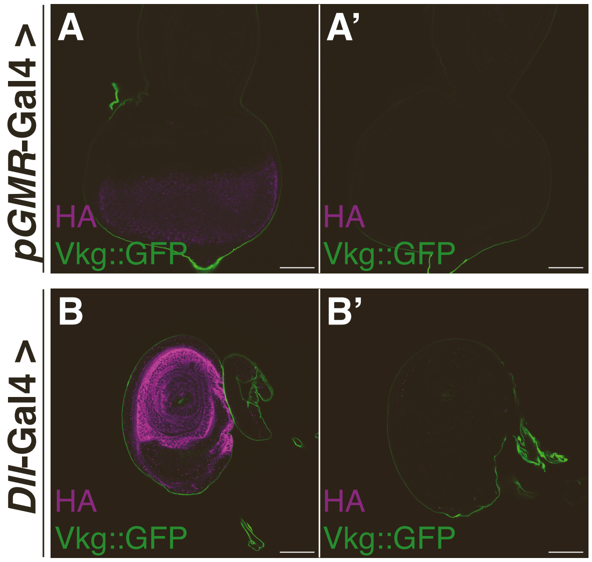

Figure S4: Knockdown of SPARC::HA in the eye or leg discs decreases HA staining intensity, related to Figure 3.

(A-B') Immunostaining of HA (magenta) in eye or leg discs expressing SPARC::HA (A and B, respectively) or SPARC::HA, *SPARC*^{RNAi} (A' and B', respectively). Vkg::GFP (green) was used to visualize *Vkg.* Scale bars represent 25 µm. Data shown are representative of a minimum of three biological replicates.

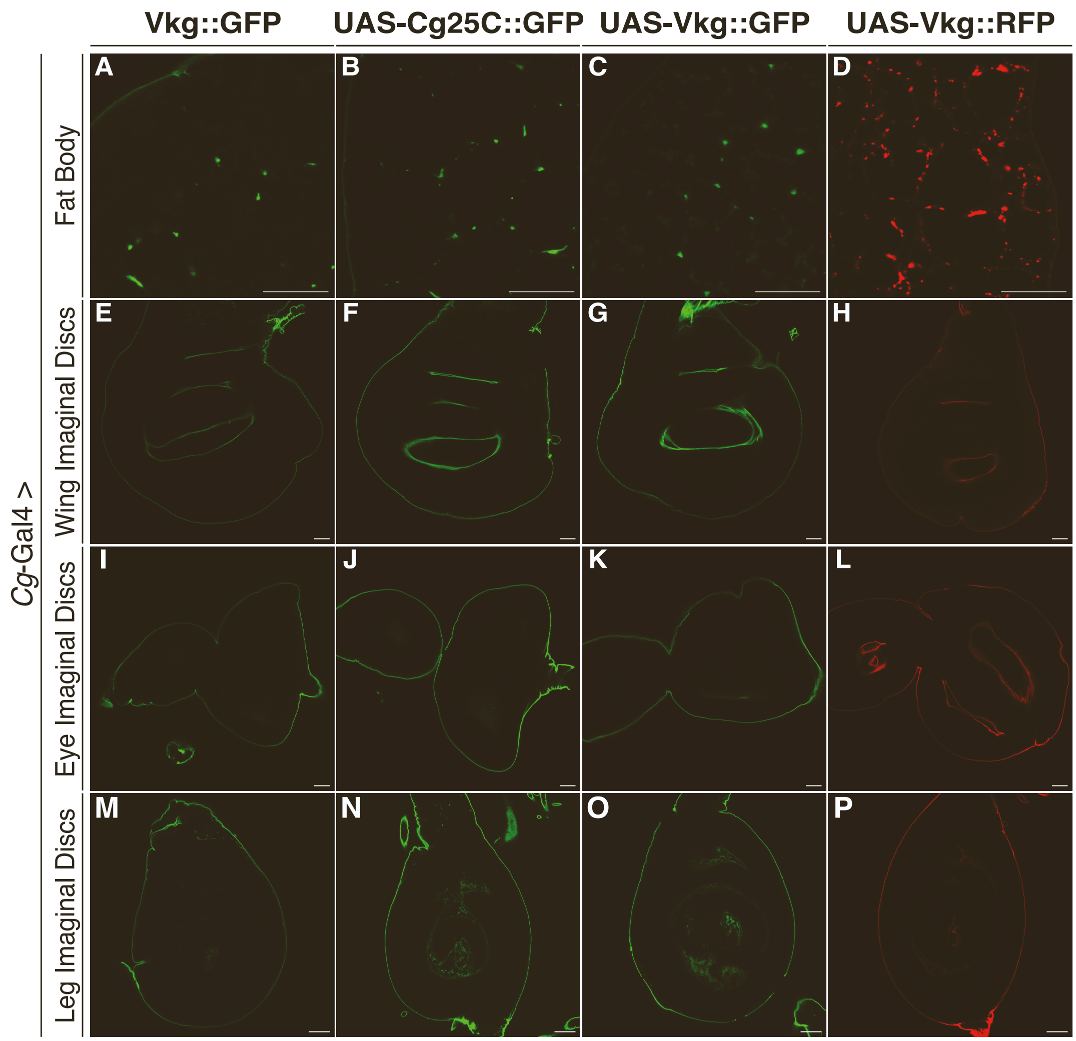

Figure S5: Fat body-derived UAS-Cg25C::GFP, UAS-Vkg::GFP, and UAS-Vkg::RFP localize to CIVICs and the BM of the fat body, wing discs, eye discs, and leg discs, related to Figure 4.

(A-D) Confocal images of third instar larval fat bodies showing the localization of endogenous Vkg::GFP (A), and fat body-derived UAS-Cg25C::GFP (B), UAS-Vkg::GFP (C), or UAS-Vkg::RFP (D). Scale bars represent 25 µm.

(E-H) Confocal images of third instar larval wing discs showing the localization of endogenous Vkg::GFP (E), and fat body-derived UAS-Cg25C::GFP (F), UAS-Vkg::GFP (G), or UAS-Vkg::RFP (H). Scale bars represent 25 µm.

(I-L) Confocal images of third instar larval eye discs showing the localization of endogenous Vkg::GFP (I), and fat body-derived UAS-Cg25C::GFP (J), UAS-Vkg::GFP (K), or UAS-Vkg::RFP (L). Scale bars represent 25 µm.

(M-P) Confocal images of third instar larval leg discs showing the localization of endogenous Vkg::GFP (M), and fat body-derived UAS-Cg25C::GFP (N), UAS-Vkg::GFP (O), or UAS-Vkg::RFP (P). Scale bars represent 25 µm.

Data shown are representative of a minimum of three biological replicates.

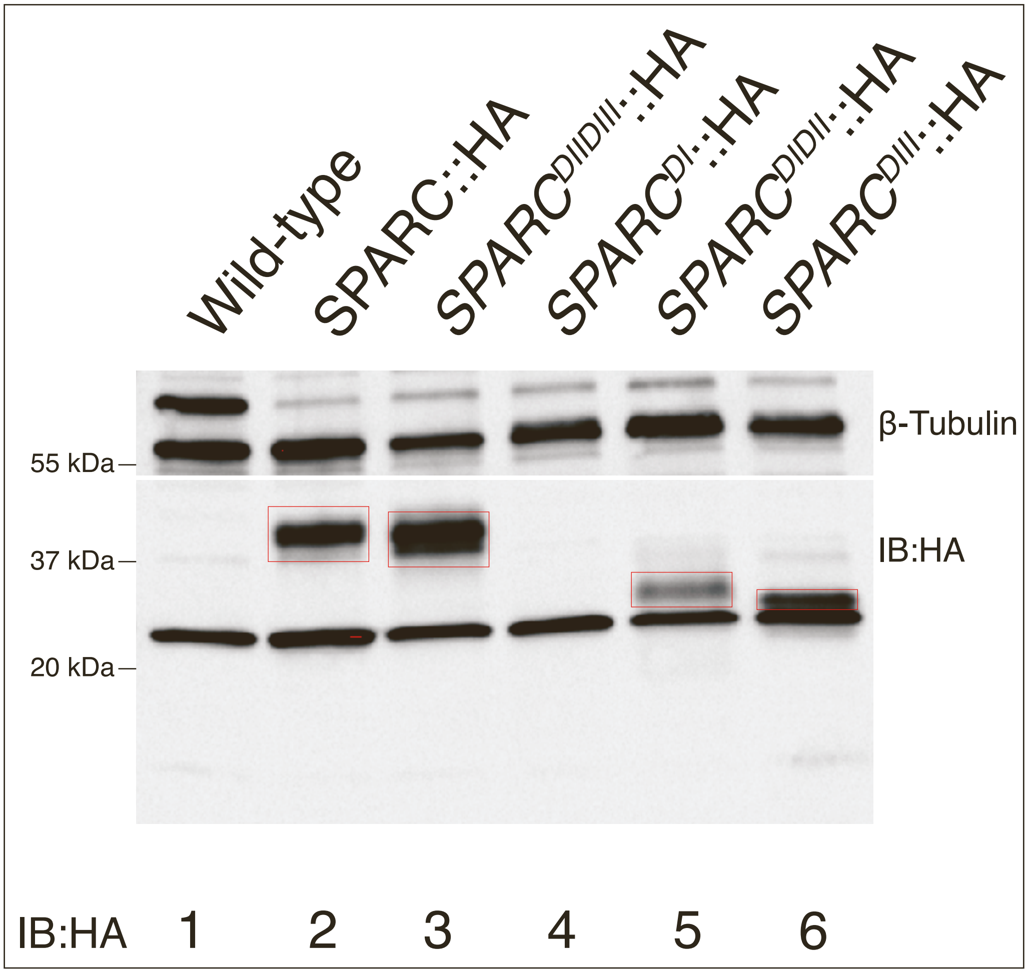

Figure S6: Expression levels of the UAS-SPARC::HA modular constructs, related to Figure 6.

Western blot analyses of UAS-SPARC::HA modular constructs being expressed. **Lane 1.** Wild-type. **Lane 2.** UAS-SPARC::HA. **Lane 3.** UAS-*SPARC*^{DIIDIII}::HA. **Lane 4.** UAS-*SPARC*^{DI}::HA. **Lane 5.** UAS-*SPARC*^{DIDII}::HA. **Lane 6.** UAS-*SPARC*^{DIII}::HA. β-tubulin (~55 kDa) was used as a loading control. Molecular weights are indicated on the left. Data shown are representative of a minimum of three biological replicates.

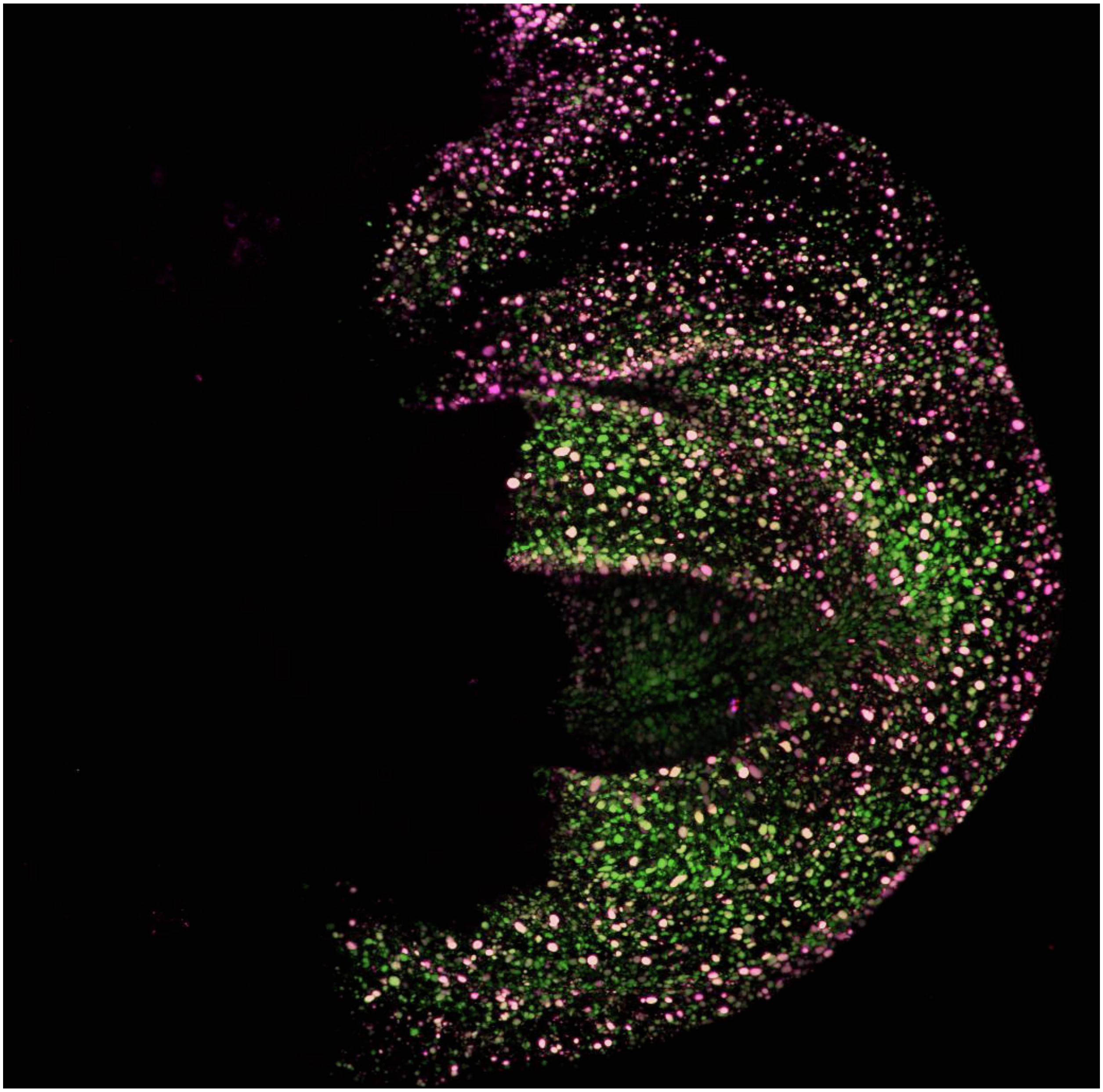

Video S1: 3D reconstruction of a third instar wing disc

This video shows a 3D reconstruction of third instar wing discs expressing UAS-Cg25C::GFP (green), UAS-Vkg::RFP (red) and UAS-SPARC::HA (magenta) and the distribution of punctae throughout the tissue.
